# Association of alcohol consumption with morbidity and mortality in patients with cardiovascular disease: original data and meta-analysis of 48,423 men and women

**DOI:** 10.1186/s12916-021-02040-2

**Published:** 2021-07-27

**Authors:** Chengyi Ding, Dara O’Neill, Steven Bell, Emmanuel Stamatakis, Annie Britton

**Affiliations:** 1 Research Department of Epidemiology and Public Health, University College London, London, UK; 2 CLOSER, Department of Social Science, Institute of Education, University College London, London, UK; 3 The National Institute for Health Research Blood and Transplant Unit in Donor Health and Genomics, University of Cambridge, Cambridge, UK; 4 British Heart Foundation Cardiovascular Epidemiology Unit, Department of Public Health and Primary Care, University of Cambridge, Cambridge, UK; 5 Stroke Research Group, Department of Clinical Neurosciences, University of Cambridge, Cambridge Biomedical Campus, Cambridge, UK; 6 Charles Perkins Centre, Sydney School of Public Health, Faculty of Medicine and Health, University of Sydney, Sydney, Australia

**Keywords:** Alcohol, Cardiovascular disease, Mortality, Secondary prevention, Meta-analysis

## Abstract

**Background:**

Light-to-moderate alcohol consumption has been reported to be cardio-protective among apparently healthy individuals; however, it is unclear whether this association is also present in those with disease. To examine the association between alcohol consumption and prognosis in individuals with pre-existing cardiovascular disease (CVD), we conducted a series of meta-analyses of new findings from three large-scale cohorts and existing published studies.

**Methods:**

We assessed alcohol consumption in relation to all-cause mortality, cardiovascular mortality, and subsequent cardiovascular events via de novo analyses of 14,386 patients with a previous myocardial infarction, angina, or stroke in the UK Biobank Study (median follow-up 8.7 years, interquartile range [IQR] 8.0–9.5), involving 1640 deaths and 2950 subsequent events, and 2802 patients and 1257 deaths in 15 waves of the Health Survey for England 1994–2008 and three waves of the Scottish Health Survey 1995, 1998, and 2003 (median follow-up 9.5 years, IQR 5.7–13.0). This was augmented with findings from 12 published studies identified through a systematic review, providing data on 31,235 patients, 5095 deaths, and 1414 subsequent events. To determine the best-fitting dose-response association between alcohol and each outcome in the combined sample of 48,423 patients, models were constructed using fractional polynomial regression, adjusting at least for age, sex, and smoking status.

**Results:**

Alcohol consumption was associated with all assessed outcomes in a J-shaped manner relative to current non-drinkers, with a risk reduction that peaked at 7 g/day (relative risk 0.79, 95% confidence interval 0.73–0.85) for all-cause mortality, 8 g/day (0.73, 0.64–0.83) for cardiovascular mortality and 6 g/day (0.50, 0.26–0.96) for cardiovascular events, and remained significant up to 62, 50, and 15 g/day, respectively. No statistically significant elevated risks were found at higher levels of drinking. In the few studies that excluded former drinkers from the non-drinking reference group, reductions in risk among light-to-moderate drinkers were attenuated.

**Conclusions:**

For secondary prevention of CVD, current drinkers may not need to stop drinking. However, they should be informed that the lowest risk of mortality and having another cardiovascular event is likely to be associated with lower levels of drinking, that is up to approximately 105g (or equivalent to 13 UK units, with one unit equal to half a pint of beer/lager/cider, half a glass of wine, or one measure of spirits) a week.

**Supplementary Information:**

The online version contains supplementary material available at 10.1186/s12916-021-02040-2.

## Background

Lifestyle and dietary habits play an important role in the secondary prevention of cardiovascular disease (CVD) [1]. However, the impact of alcohol consumption on CVD patients’ prognosis is unclear and recommendations for patients regarding upper limits of drinking vary substantially across different guidelines [2–5]. While light-to-moderate alcohol consumption is associated with a lower risk of developing multiple cardiovascular outcomes in general population cohorts [6, 7], it is difficult to extend the posited cardio-protective effects to CVD patients because of their typically older age and compromised vasculature as well as the medications they take to prevent secondary events [8]. In addition, for CVD patients, there are concerns about the potential detrimental effects of alcohol on the circulatory system, such as hypertension, arrhythmias, and haemorrhagic stroke, which may exacerbate their existing pathological conditions [9].

The most recent meta-analysis to have explored the association between alcohol consumption and prognosis among CVD patients was undertaken by Costanzo et al. in 2010 [10]. Pooling data from eight observational studies published between 1998 and 2008, they identified a maximal 22% relative risk (RR) reduction at approximately 8 g/day for cardiovascular mortality and 18% at 7 g/day for all-cause mortality among patients with myocardial infarction (MI), angina, or stroke, relative to non-drinkers, with risk increasing in a dose dependent manner above these levels. However, their analysis was limited to studies only on mortality and did not consider any non-fatal outcomes. Understanding how alcohol consumption is related to cardiovascular morbidity is of great importance to CVD patients because this population is at high risk of recurring cardiovascular events which can significantly compromise the patients’ quality of life [11]. Including morbidity information will complement the existing evidence base to provide a more complete picture of how alcohol consumption can be managed for optimal secondary CVD prevention. Additionally, further studies [12–14] have been published in the decade since the last meta-analysis. Given the growing debate on this topic, a more detailed and comprehensive reassessment of the evidence is warranted in the absence of long-term clinical trials [9].

We thus analysed individual data from three large-scale cohorts. In addition to estimating risk of mortality among CVD patients, we also examined the association between alcohol intake and subsequent cardiovascular events. To consolidate all available evidence on this topic, we conducted meta-analyses of our results with those from published studies identified through a systematic review.

## Methods

### De novo cohort analyses

#### Study cohorts and participants

Data were obtained from participants in the Health Survey for England (HSE), the Scottish Health Survey (SHeSs), and UK Biobank. Descriptions of each cohort are provided in Additional file 1 (Appendix S1). The present analyses combined data from the 1994–2008 HSE datasets and the 1995, 1998, and 2003 SHeSs datasets and were restricted to participants aged ≥16 years reporting to have been diagnosed with MI/angina (not recorded separately) or stroke prior to baseline. For UK Biobank, we identified participants with MI, angina, or stroke before recruitment based on record linkage to the Hospital Episode Statistics (HES), using algorithms defined in Additional file 1 (Appendix S1 and Table S1 [15–29]).

To be eligible for the analysis, participants in HSE/SHeSs and UK Biobank had to have baseline information about their drinking status and average alcohol intakes, plus age, sex, smoking status, self-reported history of diabetes and hypertension, socioeconomic position/education, body mass index, and regular medications. We separated former drinkers from never drinkers and categorised current drinkers into three groups: low-level drinkers (≤ 14 units/week, one unit contains 8g of ethanol [30] and is equivalent to half a pint of beer/lager/cider, half a glass of wine, or one measure of spirits/fortified wine [31]), medium-level drinkers (>14 to ≤50 units/week for men, >14 to ≤35 units/week for women), and high-level drinkers (>50 units/week for men, >35 units/week for women) [32]. Further details of the alcohol assessment and covariates are described in Additional file 1 (Appendix S1).

We assessed alcohol consumption in relation to three outcomes (each ascertained by national death registries or HES records): all-cause mortality, cardiovascular mortality, and major cardiovascular events. We defined cardiovascular events as a composite of angina, fatal and non-fatal MI and stroke, revascularisation procedures (angioplasty or coronary artery bypass graft), death from heart failure, and sudden cardiac death, and only UK Biobank contributed data to the analysis on cardiovascular events. Participants were followed up until the date of their death or first detected event, or were censored on the date they left the UK or the last date of data linkage (cohort specific). Additional details of outcome ascertainment and follow-up procedures are in Additional file 1 (Appendix S1).

#### Statistical analysis

We used multivariable Cox proportional hazard models to calculate hazard ratios (HRs) and 95% confidence intervals (CIs) for the associations of different drinking categories with each outcome of interest relative to never drinkers. Adjustments were made for age, sex, and smoking status in initial models and then for all covariates in maximally-adjusted models. For HSE/SHeSs datasets, we additionally adjusted for survey wave using shared-frailty models to account for within-group correlations. Schoenfeld residuals were plotted to ascertain that the proportional hazards assumption had not been violated (see Additional file 1: Figure S1). Models for MI, angina and stroke as primary event in further stratified analyses were adjusted for each other as well as all covariates.

### Systematic review and meta-analysis

#### Search strategy and study selection

This study followed PRISMA and MOOSE guidelines [33, 34]. MEDLINE and Embase were searched for relevant studies up to 30 July 2020, using a combination of subject headings and free-text terms with no restrictions on language or publication date (see Additional file 1: Table S2). In addition, the reference lists of eligible studies and a previous systematic review [35] on this topic were manually checked to add any studies missed by the initial database searches.

After removing duplicates, citations were screened to exclude any that did not report a prospective relationship between alcohol consumption and outcomes of interest among patients with pre-existing CVD. Full text of the remaining citations were then independently assessed by two pairs of reviewers (CD and AB/DON) for eligibility. Studies were retained if they met the selection criteria for study design (longitudinal study including randomised control trials not involving alcohol), study population (MI, angina, or stroke patients), exposure (alcohol consumption reported across ≥3 categories, inclusive of a non-drinking group, to allow for testing a curvilinear relationship), outcomes (all-cause or cardiovascular mortality, cardiovascular events), and risk estimates (at least adjusted for age, sex, and smoking). We excluded studies if the reported alcohol consumption could not be converted into gram per day or if frequency counts, risk estimate, and its corresponding 95%CI were not available after contacting the authors. The inter-rater agreement for this review was high (Fleiss κ= 0.85).

#### Data extraction and quality assessment

Data extraction was conducted by one reviewer (CD) and then verified by a second reviewer (AB/DON). When available, we collected data on the amount of alcohol consumed. Given that most studies included in our analyses reported alcohol consumption on a daily basis, we used grams of alcohol per day as the common unit of measurement. To convert the number of drinks to grams in four included studies (one conducted in Italy [12] and three in USA [36–38]) which did not specify the quantity of alcohol in one drink, we assumed country-specific standard drinks (i.e. Italy 12g, USA 14g) [39]. A factor of 0.79 was used for the conversion of millilitres to grams (i.e. 1 ml alcohol = 0.79 g [40]) in one study [41]. Exposures categorised according to time periods longer than 1 day were transferred into daily estimates, assuming an even distribution of consumption over the reference period. Where averages were not reported for each drinking category, the midpoints of the range were chosen. For open-ended upper categories, mean values were defined as 1.2 times the lower boundary as suggested by Berlin et al. [42]. Similar results were obtained when multiplying the lower boundary for the open-ended upper categories by 1.0, 1.4, or 1.6 instead of 1.2 (see Additional file 1: Figure S2).

Multiple alcohol measures were used in three included studies, two of which reported risk estimates based on the average intakes during follow-up [13, 43] and the remaining one performed time-dependent analyses to allow changes on drinking habits [12]. In addition, most of the included studies asked patients to report their average consumption since the occurrence of their primary events (post-event alcohol assessment), whereas three studies used alcohol intake in the year prior to primary events (pre-event), assuming drinking habits remained stable over time, even following events [14, 44, 45].

Because all included studies except one [46] used a non-drinking reference group, we preferred risk estimates for different drinking categories versus non-drinkers. For a single study that used occasional drinkers as the reference group [46], the risk estimates were recalculated to derive alternative estimates each relative to a non-drinker group. A Microsoft Excel spreadsheet developed by Hamling et al. was used during the recalculation to account for the non-independence between estimates sharing a common reference group [47]. When a study reported risk estimates with different degrees of statistical adjustment for confounding, we used the most-adjusted one. Furthermore, to investigate the possible impact of over-adjustment for potential mediators on our results, we performed a sensitivity analysis by using risk estimates that were only controlled for age, sex, and smoking, the three most important confounding factors for the alcohol-CVD relationship. With all estimates reported being RR or HR, RR served as the common measure of association across studies. HRs were treated as measures of RRs [48]. Study quality was assessed using the Newcastle-Ottawa Scale (see Additional file 1: Appendix S2) [49].

#### Data synthesis

For each analysis, a family of second-degree fractional polynomial models (FP2: log RR = β_1_x^p1^ +β_2_x^p2^, x^0^ equals log(x) rather than 1 and the model becomes log RR = β_1_x^p^ + β_2_x^p^log(x) when p_1_ = p_2_) was generated to derive a power transformation of the exposure variable [50]. p_1_ and p_2_ were taken from a predefined set P= (−2, −1, −0.5, 0, 0.5, 1, 2, 3) which allows for a very large and varied set of functions, including U- and J-shaped curves, to be generated. For x = 0, the function would start from log RR = 0 and therefore no constant term (i.e. the intercept) was considered in our models [51]. The best fit among the family of models was defined as that with the lowest deviance.

With the terms of exposure identified in the best-fitting FP2, a two-stage regression model was fitted to summarise the relationship between alcohol consumption and each outcome of interest. The first stage generated the dose-response model within each study and the second stage pooled study-specific trends using a random effect model to accommodate the heterogeneity across studies [52, 53]. A sensitivity analysis was done by excluding studies of the lowest quality and pre-defined subgroup analyses according to sex, primary event, and type of non-drinking reference group and alcohol assessment for each outcome of interest.

The overall degree of heterogeneity was quantified using the *I*^*2*^ index [54]. We assessed evidence of publication bias through visual inspection of funnel plots and Egger’s regression test for asymmetry [55]. All statistical analyses were performed using Stata (version 15.1).

## Results

### Associations of alcohol consumption with mortality and cardiovascular morbidity in study cohorts

Complete data for the de novo cohort analyses were available for 2802 participants (MI/angina=2341, stroke=535) in HSE/SHeSs and 14,386 (MI=5333, angina=9589, stroke=2064) in UK Biobank (see Additional file 1: Figure S3). On average, UK Biobank participants were younger and reported higher consumption of alcohol than HSE/SHeSs participants (Table 1).

**Table 1:** Characteristics of participants at baseline

	HSE/SHeSs						UK Biobank					
	Never drinker	Former drinker	Low-level drinker	Medium-level drinker	High-level drinker	Overall	Never drinker	Former drinker	Low-level drinker	Medium-level drinker	High-level drinker	Overall
N	263 (9.4)	383 (13.7)	1630 (58.2)	458 (16.3)	68 (2.4)	2802 (100.0)	1076 (7.5)	1207 (8.4)	5989 (41.6)	5222 (36.3)	892 (6.2)	14386 (100.0)
Age, mean (SD), y	69.0 (11.0)	67.1 (11.1)	68.2 (10.1)	64.2 (10.9)	60.4 (10.7)	67.3 (10.6)	61.6 (6.6)	61.1 (6.5)	61.9 (6.1)	61.6 (6.0)	60.5 (6.4)	61.6 (6.2)
Alcohol intake, mean (SD), g/day	0.0	0.0	4.0 (4.3)	28.0 (10.2)	85.1 (33.0)	9.0 (16.9)	0.0	0.0	7.9 (5.1)	30.6 (10.6)	76.7 (26.4)	19.2 (21.4)
BMI, mean (SD), kg/m^2^	28.5 (5.5)	28.6 (5.7)	27.9 (4.6)	27.9 (4.1)	28.0 (4.2)	28.1 (4.8)	30.0 (5.8)	30.2 (6.0)	29.1 (5.0)	28.8 (4.3)	29.0 (4.7)	29.2 (4.9)
Female	187 (71.1)	189 (49.3)	758 (46.5)	65 (14.2)	5 (7.4)	1204 (43.0)	619 (57.5)	447 (37.0)	2242 (37.4)	743 (14.2)	174 (19.5)	4225 (29.4)
Smoking status												
Never	157 (59.7)	89 (23.2)	527 (32.3)	78 (17.0)	10 (14.7)	861 (30.7)	704 (65.4)	350 (29.0)	2616 (43.7)	1512 (29.0)	178 (20.0)	5360 (37.3)
Ex-smoker	66 (25.1)	191 (49.9)	799 (49.0)	272 (59.4)	29 (42.6)	1357 (48.4)	252 (23.4)	638 (52.9)	2799 (46.7)	3045 (58.3)	507 (56.8)	7241 (50.3)
Current smoker	40 (15.2)	103 (26.9)	304 (18.7)	108 (23.6)	29 (42.6)	584 (20.8)	120 (11.2)	219 (18.1)	574 (9.6)	665 (12.7)	207 (23.2)	1785 (12.4)
History of diabetes	40 (15.2)	74 (19.3)	169 (10.4)	43 (9.4)	2 (2.9)	328 (11.7)	280 (26.0)	346 (28.7)	1026 (17.1)	676 (12.9)	117 (13.1)	2445 (17.0)
History of hypertension	50 (19.0)	60 (15.7)	224 (13.7)	54 (11.8)	6 (8.8)	394 (14.1)	637 (59.2)	764 (63.3)	3193 (53.3)	2940 (56.3)	536 (60.1)	8070 (56.1)
Socioeconomic position^a^												
Low	106 (40.3)	186 (48.6)	764 (46.9)	230 (50.2)	26 (38.2)	1312 (46.8)	NA	NA	NA	NA	NA	NA
Intermediate	104 (39.5)	138 (36.0)	494 (30.3)	83 (18.1)	24 (35.3)	843 (30.1)	NA	NA	NA	NA	NA	NA
High	53 (20.2)	59 (15.4)	372 (22.8)	145 (31.7)	18 (26.5)	647 (23.1)	NA	NA	NA	NA	NA	NA
Highest educational qualification^b^												
None	NA	NA	NA	NA	NA	NA	432 (40.1)	564 (46.7)	1910 (31.9)	1510 (28.9)	247 (27.7)	4663 (32.4)
O levels or equivalent	NA	NA	NA	NA	NA	NA	141 (13.1)	150 (12.4)	900 (15.0)	742 (14.2)	149 (16.7)	2082 (14.5)
A levels or equivalent	NA	NA	NA	NA	NA	NA	315 (29.3)	295 (24.4)	1948 (32.5)	1760 (33.7)	305 (34.2)	4623 (32.1)
Degree	NA	NA	NA	NA	NA	NA	188 (17.5)	198 (16.4)	1231 (20.6)	1210 (23.2)	191 (21.4)	3018 (21.0)
Cholesterol-lowering medications	70 (26.6)	128 (33.4)	328 (20.1)	107 (23.4)	11 (16.2)	644 (23.0)	841 (78.2)	990 (82.0)	4876 (81.4)	4488 (85.9)	732 (82.1)	11927 (82.9)
Antihypertensive medications	168 (63.9)	247 (64.5)	883 (54.2)	217 (47.4)	27 (39.7)	1542 (55.0)	746 (69.3)	855 (70.8)	4047 (67.6)	3774 (72.3)	651 (73.0)	10073 (70.0)
Antiplatelet agents	118 (44.9)	187 (48.8)	725 (44.5)	207 (45.2)	23 (33.8)	1260 (45.0)	810 (75.3)	902 (74.7)	4655 (77.7)	4305 (82.4)	736 (82.5)	11408 (79.3)
Digoxin	9 (3.4)	19 (5.0)	62 (3.8)	10 (2.2)	2 (2.9)	102 (3.6)	16 (1.5)	29 (2.4)	86 (1.4)	66 (1.3)	12 (1.3)	209 (1.5)
Warfarin^c^	NA	NA	NA	NA	NA	NA	59 (5.5)	106 (8.8)	358 (6.0)	313 (6.0)	34 (3.8)	870 (6.0)

Data are number (percentage) unless otherwise specified

*BMI*, body mass index; *HSE*, the Health Survey for England; *NA*, not applicable; *SD*, standard deviation; *SHeSs*, the Scottish Health Survey

^a^Socioeconomic position was defined using the participant’s occupational classification, categorised as low (semi-skilled or unskilled manual), intermediate (skilled non-manual or manual), or high (professional or managerial technical)

^b^Highest educational qualification was categorised into four levels: None, O levels/GCSEs, CSEs or equivalent; A/AS levels, NVQ or HND or HNC or equivalent, or other professional qualification; college or university degree

^c^None of the participants in HSE/SHeSs reported using warfarin on a regular basis

**Fig. 1:**
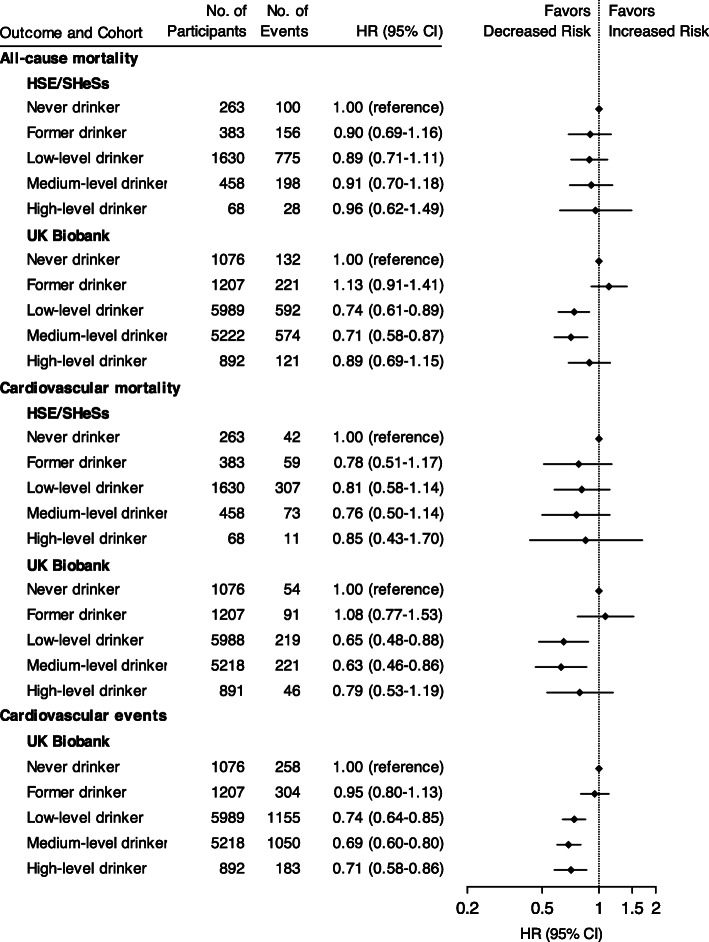
Association of drinking categories with all-cause mortality, cardiovascular mortality, and cardiovascular events by study cohorts. Hazard ratios are adjusted for age, sex, smoking status, diabetes, hypertension, socioeconomic position or education, body mass index, and regular use of cholesterol-lowering medications, antihypertensive medications, antiplatelet agents, digoxin, and warfarin. CI indicates confidence interval; HR, hazard ratio; HSE, the Health Survey for England; SHeSs, the Scottish Health Survey

During a median follow-up of 9.5 years (interquartile range [IQR], 5.7–13.0) in HSE/SHeSs and 8.7 years (IQR, 8.0–9.5) in UK Biobank, we identified 1257 deaths among HSE/SHeSs participants and 1640 deaths among UK Biobank participants, of which 492 (39.1%) and 631 (38.5%) deaths were due to cardiovascular causes, respectively. Maximally adjusted models of UK Biobank dataset revealed a J-shaped association for both all-cause and cardiovascular mortality, with low- and medium-level drinkers having a decreased risk compared with never drinkers but no difference in risk for high-level or former drinkers (Fig. 1). Although similar J-shaped trends were observed for HSE/SHeSs, none of the associations were statistically significant, probably due to the relatively small sample size of each drinking subgroup (Fig. 1). We noted differential associations by sex and primary cardiovascular events in stratified analyses (see Additional file 1: Figures S4 and S5).

A total of 2950 fatal and non-fatal subsequent cardiovascular events were recorded in UK Biobank, with a median follow-up of 7.5 years (IQR, 6.8–8.5). A lower risk of cardiovascular events was observed across all categories of current drinkers (Fig. 1), within participants of both sexes and with different primary events (see Additional file 1: Figures S4 and S5).

### Characteristics of studies included in meta-analysis

Of the initial 1722 unique citations, 12 published studies fulfilled the selection criteria (see Additional file 1: Figure S6). Table 2 outlines the characteristics of all studies selected for meta-analyses, inclusive of HSE/SHeSs and UK Biobank. Nine of the 14 studies had a cohort design and the remaining five [12, 36–38, 43] were randomised control trials for certain drug or diet type with no specific inventions on alcohol consumption. The quality of selected studies was moderate to high on average, with a median score of 8 on the Newcastle-Ottawa Scale. Additional details regarding alcohol consumption, effect estimates, and confounder adjustment are provided in Additional file 1 (Tables S3–S5).

**Table 2:** Characteristics of 14 studies included in meta-analyses

Source	Country	Dataset	Sex	Study size, No.	Meta-analyses Inclusion^a^			Follow up, y^b^	Baseline age, y^c^	Reference group including former drinkers	Pre-/post-event alcohol assessment	Multiple alcohol measures	Quality assessment score	Primary event
					ACM case, No.	CVM case, No.	CVE case, No.							
HSE/SHeSs	UK	HSE (1994–2008) / SHeSs (1995, 1998, 2003)	M, F	2802	1257	492	NA	9.5	67.3	Both^d^	Post-	No	9	MI/angina, stroke^e^
UK Biobank	UK	Initial assessment visit (2006–2010)	M, F	14,386	1640	631	2950	8.7	61.6	Both^d^	Post-	No	9	MI, angina, stroke
Levantesi et al., 2013 [12]	Italy	GISSI study	M, F	11,248	1656	NA	1168	5.7	59.4	Yes	Post-	Yes	7	MI
Pai et al., 2012 [13]	USA	Health Professionals Follow-up Study	M	1818	468	243	NA	Up to 20	Range 40–75	Yes	Both	Yes	7	MI
Rosenbloom et al., 2012 [14]	USA	Onset study	F	1253	441	NA	NA	Up to 10	66.1	Yes	Pre-	No	9	MI
Janszky et al., 2008 [44]	Sweden	SHEEP study	M, F	1332	259	140	NA	8.6	59.4	No	Pre-	No	9	MI
Masunaga et al., 2006 [41]	Japan	Consecutive patients	M	3845	NA	NA	142	1.1	57.2	No	Post-	No	8	MI
Aguilar et al., 2004 [36]	USA, Canada	SAVE trial	M, F	2036	355	284	NA	3.5	59.2	Yes	Both	No	7	MI
Jackson et al., 2003 [37]	USA	Physicians’ Health Study	M	1320	369	267	NA	4.5	67.4	Yes	Post-	No	6	Stroke
de Lorgeril et al., 2002 [43]	France	Lyon Diet Heart Study	M	353	NA	NA	104	4.0	54.0	Yes	Post-	Yes	7	MI
Mukamal et al., 2001 [45]	USA	Onset study	M, F	1913	317	238	NA	3.8	61.8	Yes	Pre-	No	8	MI
Shaper et al., 2000 [46]	UK	British Regional Heart Study	M	596	258	184	NA	12.8	Range 45–64	No	Post-	No	9	MI, angina
Valmadrid et al., 1999 [56]	USA	WESDR	M, F	163	NA	52	NA	Up to 12.3	68.6	No	Post-	No	9	MI/angina^e, f^
Muntwyler et al., 1998 [38]	USA	Physicians’ Health Study	M	5358	920	NA	NA	5.0	64.1	Yes	Post-	No	6	MI

*ACM* all-cause mortality, *CVE* cardiovascular events, *CVM* cardiovascular mortality, *F* female, *HSE* the Health Survey for England, *M* male, *MI* myocardial infarction, *SHeSs* the Scottish Health Survey

^a^Not applicable (NA) if the study was not included in meta-analysis on the outcome

^b^Data are mean/median unless otherwise specified

^c^Data are mean unless otherwise specified

^d^Former drinkers were included only in subgroup meta-analyses on different non-drinking reference group

^e^Results were not reported separately for angina and MI patients

^f^Older-onset diabetic patients with a history of angina or MI

### Alcohol consumption and all-cause mortality among CVD patients

**Fig. 2:**
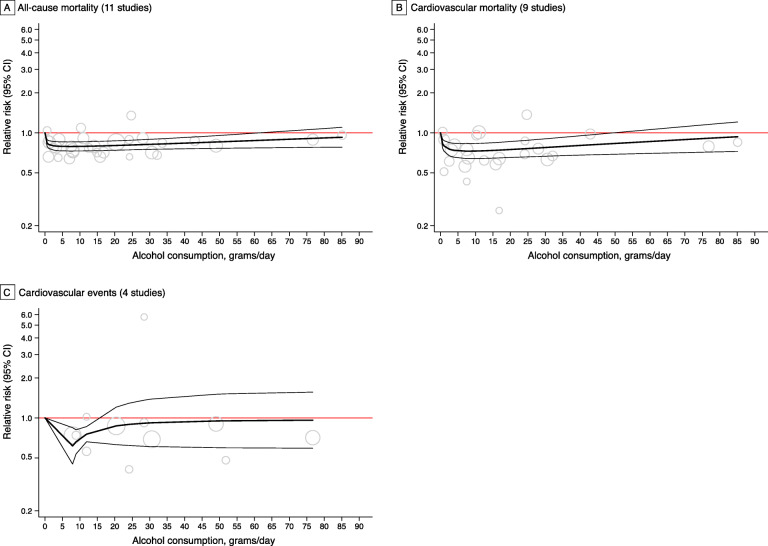
Overall dose-response relationship between alcohol consumption and risk of mortality and subsequent cardiovascular events, using maximally-adjusted estimates. Best-fitting second-degree fractional polynomial models (with 95% CIs) are shown in solid curves with each data point overlaid as circles. Circle size indicates the weighting of each data point and is inversely proportional to the variance of the log-transformed relative risk

Eleven studies, comprising 41,743 CVD patients, contributed to this analysis. Overall, a J-shaped association was observed, with a protective effect that peaked at 7 g/day and remained significant up to 62 g/day (Fig. 2A, Table 3). Although the dose-response trend followed a J-curve in men, we found no increased risk among women at higher levels of drinking (see Additional file 1: Figure S7). Regarding primary events, moderate drinking was associated with a lower risk for total mortality among patients with a previous MI or angina, but not with stroke (see Additional file 1: Figure S8). Pooled analysis of estimates relative to non-current drinkers showed a reduced mortality risk for an alcohol intake up to approximately 75 g/day. However, when studies with former drinkers in the reference group were excluded, the association was considerably weakened (see Additional file 1: Figure S9). In addition, among those studies using post-event alcohol measures, the result did not change substantively; a similar trend was seen in studies with multiple measures but failed to reach statistical significance, probably because of the low number of curves (n=2) in this subgroup (see Additional file 1: Figure S10).

**Table 3:** Best-fitting models and results of the meta-analysis on alcohol consumption and risk of mortality and subsequent cardiovascular events

Outcome and subgroup	No. of studies (curves)	No. of patients	Maximal effect size^a^		Reversion point, g/day^b^	Powers for the Best-Fitting FP2	
			RR (95% CI)	g/day		dose_1	dose_2
**All-cause mortality**							
Overall	11 (11)	41,743	0.79 (0.73–0.85)	7	62	−0.5	1
Male	6 (6)	19,897	0.82 (0.72–0.93)	9	39	0	0.5
Female	3 (3)	6046	0.64 (0.36–1.14)	54	49	−2	3
MI as primary event	9 (9)	29,554	0.82 (0.68–0.99)	2	7	−1	0.5
Angina as primary event	2 (2)	8938	0.79 (0.63–0.99)	39	46	0.5	3
Stroke as primary event	3 (3)	3618	0.71 (0.42–1.20)	12	NA	0	0.5
Reference group including former drinkers	9 (9)	41,405	0.77 (0.69–0.85)	16	75	−0.5	2
Reference group excluding former drinkers	4 (4)	17,526	0.85 (0.71–1.00)	3	3	−0.5	−0.5
Post-event alcohol assessment	8 (8)	37,245	0.81 (0.74–0.88)	9	52	0	0.5
Multiple alcohol measures	2 (2)	12,337	0.78 (0.59–1.03)	16	NA	−0.5	−0.5
**Cardiovascular mortality**							
Overall	9 (9)	24,770	0.73 (0.64–0.83)	8	50	0	0.5
Male	5 (5)	14,536	0.72 (0.62–0.85)	9	32	0	0.5
Female	2 (2)	4790	0.29 (0.09–1.01)	54	54	0	2
MI as primary event	6 (6)	12,422	0.76 (0.64–0.91)	3	25	−2	3
Angina as primary event	2 (2)	8934	0.72 (0.42–1.23)	56	NA	3	3
Stroke as primary event	3 (3)	3617	0.63 (0.37–1.08)	26	NA	0	3
Reference group including former drinkers	6 (6)	24,269	0.73 (0.58–0.93)	13	27	0	0.5
Reference group excluding former drinkers	5 (5)	17,683	0.71 (0.55–0.90)	7	29	−0.5	0.5
Post-event alcohol assessment	7 (7)	21,525	0.73 (0.60–0.90)	8	43	0	0
Multiple alcohol measures	1 (1)	1818	0.58 (0.40–0.84)	17	33	−0.5	3
**Cardiovascular events**							
Overall^c^	4 (5)	28,621	0.50 (0.26–0.96)	6	15	−2	−2
Male	3 (4)	13,598	0.56 (0.23–1.34)	8	NA	−2	−2
Female	1 (1)	3775	0.67 (0.43–1.05)	54	49	−2	3
MI as primary event	4 (5)	20,361	0.79 (0.66–0.94)	11	35	−2	3
Angina as primary event	1 (1)	8747	0.69 (0.59–0.81)	35	n.a.	−2	1
Stroke as primary event	1 (1)	1855	0.49 (0.26–0.92)	72	n.a.	−2	3
Reference group including former drinkers	3 (3)	25,983	0.72 (0.53–0.97)	40	45	1	1
Reference group excluding former drinkers	2 (3)	17,020	0.78 (0.46–1.31)	17	NA	3	3
Multiple alcohol measures	1 (1)	353	0.32 (0.14–0.71)	38	n.a.	2	3

*FP2* second-degree fractional polynomial model, *MI* myocardial infarction

^a^Defined as the lowest point of the dose-response curve within the range of dose reported by the studies

^b^Defined as the dose of alcohol at which protection against the outcome is no longer statistically significant at the 95% confidence level; not applicable (NA) if non-significant association was found at any level of consumption; not available (n.a.) if the association remained significant within the range of dose reported by the studies

^c^All of the four studies measured post-event alcohol consumption and had a quality score ≥ 7

### Alcohol consumption and cardiovascular mortality among CVD patients

Nine studies, comprising 24,770 patients, were included in the meta-analysis on cardiovascular mortality, and the overall association with alcohol consumption was interpreted as a J-curve. The maximal reduction in mortality risk was found to be 27% at 8 g/day and the reversion point was reached at 50 g/day (Fig. 2B, Table 3). Our results remained little altered when considering studies on men only, or using different types of reference groups or alcohol assessments (see Additional file 1: Figures S7, S9 and S10). Unlike the J-curve observed for men, there was no excess risk of mortality among women at higher levels of consumption (see Additional file 1: Figure S7). Stratified analyses by primary events showed that moderate drinking was associated with a lower risk of cardiovascular mortality among patients with a previous MI; however, among those with angina or stroke, the overall dose-response trend was close to null (see Additional file 1: Figure S8).

### Alcohol consumption and cardiovascular events among CVD patients

Among the four studies (28,621 patients) addressing drinking and cardiovascular events, one reported dose-response trend separately for two age groups and thus provided two curves. Alcohol intake was associated with a significant reduction in the risk of cardiovascular events up to 15g/day (Fig. 2C, Table 3). Pooled analysis of studies on women showed a declined risk for an alcohol intake up to approximately 49 g/day, whereas no reduction in risk was seen in men at any level of consumption (see Additional file 1: Figure S7). Moderate drinking was found to be protective against cardiovascular events within patients of different primary events and studies with multiple alcohol measures (see Additional file 1: Figures S8 and S10). However, when studies including former drinkers in the reference group were excluded, the overall protective effect was attenuated and became non-significant (see Additional file 1: Figure S9).

### Sensitivity analyses

Sensitivity analyses excluding studies of the lowest quality (score <7) revealed similar curves (see Additional file 1: Figure S11). Results were consistent when restricting analysis to estimates that were only adjusted for age, sex, and smoking status (see Additional file 1: Figure S12). For mortality outcomes, there was no evidence of heterogeneity across the first- and second-order polynomial (both *I*^*2*^ = 0%); however, a high degree of heterogeneity (both *I*^*2*^ = 75%) was noted in studies contributing results for cardiovascular events. For all outcomes assessed, we found no evidence of publication bias (see Additional file 1: Figure S13).

## Discussion

Meta-analysis of the results from three major UK cohorts together with those from 12 published studies found J-curve relationships between alcohol consumption and mortality in those with cardiovascular disease, with the greatest risk reduction being observed at 7 g/day for all-cause mortality and 8 g/day for cardiovascular mortality relative to current non-drinkers. This dose-response trend remains consistent with the last published meta-analysis [10] and has also been reported in other high-risk populations, such as hypertensive [57] and diabetic individuals [58].

To our knowledge, this is the first meta-analysis of alcohol consumption and any subsequent cardiovascular events in patients with previous CVD, in which UK Biobank contributed nearly half of the total sample size. We found a reduction in risk for an alcohol intake up to approximately 15 g/day, an upper limit much lower than those for the mortality outcomes. Taken together, our study suggested that, among CVD patients, the upper drinking limit for lower risks of mortality and cardiovascular morbidity was about 105 g/week, which was lower than those recommended in most current guidelines. For example, the American Heart Association (AHA) and American College of Cardiology Foundation 2011 guidelines on secondary prevention recommend “alcohol moderation”—up to 196 g/week (2 USA drinks/day) for male and 98 g/week (1 USA drink/day) for female according to the national dietary guidelines [59]—for patients with atherosclerotic vascular disease [2]; the same recommendations apply in the AHA/American Stroke Association 2014 guidelines for secondary stroke prevention [5]; the UK National Institute for Health and Care Excellence 2020 guidelines recommend to keep alcohol intake within 112 g/week (14 UK units/week) for both men and women after having an MI [4]; and WHO 2007 recommendations for prevention of recurrent MI and stroke were no more than about 166 g/week (3 units/day, 1 unit contains 10 ml of pure alcohol) [3].

### Strengths and limitations of study

With almost triple the number of CVD patients, our study expands the findings of the last comprehensive review published a decade ago [10]. In particular, both HSE/SHeSs and UK Biobank provide long-term follow-up of large contemporary samples from the UK general population. The inclusion of these new datasets allows us to examine the risk of drinking within various subgroups, some of which are not available or too small to reliably investigate in published studies. For example, our data suggest that the dose-response associations of alcohol with mortality and morbidity differ by sex and are more pronounced among patients with MI than angina or stroke. These findings raise the question of whether differential drinking limits should be recommended in patient subgroups and warrant further investigation. Furthermore, there is evidence that reductions in risk of all-cause mortality and subsequent events might have been overestimated due to the inclusion of former drinkers in the non-drinking reference group. Former drinkers may include individuals who have quit drinking in response to ill health (i.e. “sick quitters”), particularly past heavy drinkers [60], therefore making current drinkers appear healthy relative to less healthy non-current drinkers. This could lead to a low-risk drinking limit less than the estimated 105g/week; however, we cannot definitely determine the extent of this overestimation with very few studies that explicitly excluded former drinkers.

Many medications commonly used by CVD patients can interact with alcohol by altering the metabolism or effects of the medication and/or alcohol [61]. The interactions may occur with lower amounts of alcohol or follow a dose-response relationship, with the risk and severity of interactions increasing with increasing levels of alcohol consumption [62]. For example, moderate drinking in combination with statins use may be synergistic to confer a lower risk of all-cause mortality [63]. Concurrent heavy drinking with warfarin enhance the anticoagulant effect and may lead to major bleeding [64]. In the present meta-analyses, most (9 out of 14) but not all included studies adjusted for medication use (including antihypertensives, cholesterol-lowering and oral antiplatelet agents) in their most-adjusted models and so there is a possibility of residual confounding by medications. However, sensitivity analyses showed consistent results when using risk estimates that were only adjusted for age, sex and smoking, suggesting that further adjustment for medication use is unlikely to materially impact on our findings.

In the present study, no elevated risk of mortality and cardiovascular events was found at higher levels of alcohol consumption, which is in line with other meta-analysis among CVD patients [10, 65] but contradicts evidence from some of the general population studies [66, 67]. The discrepancy between the present study and previous general population studies may be partly due to the generally older age of CVD patients. The mean/median age at baseline was greater than 59 years in most datasets used in our analyses. Because alcohol-related risk is relatively higher among younger people compared with the elderly [68], enrolling older participants in studies would minimise the risk relationship compared with an analysis that included drinkers of all ages. Notably, with older age of the study participants comes increased likelihood for drinkers to become former drinkers, which might exacerbate the “sick quitters” bias (i.e. when the non-drinking reference group also includes former drinkers who have stopped drinking due to poor health) as discussed above. Patients who drink heavily and enrolled in studies at older ages are more likely to represent “healthy survivors” or have safer drinking patterns [60, 69]. Particularly heavy drinkers are known to be under-represented in some datasets used in our analyses, such as the Physicians’ Health Study [70] and HSE/SHeSs [71]. These potential selections may have biased downwards the estimated associations between heavy intake and risks of mortality and subsequent events. Furthermore, most included studies did not capture the extremes of drinking and therefore may be underpowered to look at the effects of very heavy drinking. Consequently, the absence of effects at higher levels of consumption seen in our study should be interpreted cautiously, particularly in light of the increasing concerns about alcohol misuse among older people [72] as well as the known wider health and societal impacts in regard to these [73].

The present study has some further limitations. First, as a composite of cardiac mortality and several non-fatal cardiovascular endpoints, the definition of cardiovascular events varied across the three published studies [12, 41, 43], and thus, we defined the outcome in UK Biobank using the most frequently reported events in these studies. However, there was still a significant heterogeneity in the pooled analysis. Recent observational and genetic evidence has suggested that drinking at moderate levels is associated with a decreased risk of some but not all forms of CVD [6, 74–76]. Therefore, this heterogeneity might have reflected the complex and diverse impacts of alcohol consumption on different CVD outcomes.

Secondly, our results must be interpreted with caution when it comes to some subgroups that have been examined in only a limited number of studies. Although the included studies scored as moderate-to-high quality on the Newcastle-Ottawa Scale, this may not account for some pertinent design/reporting characteristics of many of the studies which had problems that were specific to alcohol exposure and not covered in the scale. For example, by relying upon only a single measurement of alcohol consumption, some studies did not consider the effect of temporal changes in drinking behaviour both after primary event and during follow-up; however, our results remained consistent in the analyses restricted to studies using post-event or multiple measures. Further analyses for beverage type were not possible with sufficient beverage-specific data reported in very few studies.

Thirdly, episodic heavy drinking has been suggested to modify the relationship between average alcohol consumption and CVD/mortality risk [77]. Our results might have been confounded by the drinking pattern, as the selected studies did not exclude “binge” drinkers. Additionally, as with all observational studies and self-reported alcohol intake, our findings are prone to bias; however, self-reported drinking data was validated against high-density lipoprotein cholesterol and gamma-glutamyl transferase in HSE/SHeSs and UK Biobank (see Additional file 1: Table S6). Although we attempted to minimise confounding by using the most adjusted estimates, information on dietary habits or physical activity was not available in all studies included in our meta-analysis and residual confounding may still persist.

## Conclusions

In summary, our study shows that an alcohol intake up to about 105g (or equivalent to 13 UK units, with one unit equal to half a pint of beer/lager/cider, half a glass of wine, or one measure of spirits) a week is associated with lower risks of both mortality and subsequent cardiovascular events among CVD patients. While this threshold is somewhat lower than those recommended in most current guidelines, specific recommendations regarding the downward revision of such guidelines cannot be made. There is some indication that reductions in risk may have been overestimated by studies using a referent group contaminated by less healthy former drinkers. No evidence of elevated risk among heavy drinkers was found but this was potentially attributable to selections and under-representation of such drinkers in the datasets. Moreover, when developing drinking thresholds for use within guidelines, we must consider the totality of evidence and balance pragmatic concerns [78]. Our findings therefore indicate that, for secondary prevention of CVD, current drinkers may not need to stop drinking but should be informed that lower levels of intake (up to 105g/week) may be associated with reduced risks. However, non-drinking patients should not be encouraged to take up light drinking because of well-known adverse effects on other health outcomes, such as cancers [79].

## Supplementary Information

**Additional file 1: Supplementary Materials.** Supplementary methods for de novo cohort analyses (**Appendix S1** and **Table S1**). Quality assessment checklist (**Appendix S2**). Literature search strategy (**Table S2**). Alcohol consumption, effect estimates, and confounder adjustment reported by studies selected for meta-analyses (**Table S3-S5**). Associations of alcohol intake with HDL-cholesterol and gamma-glutamyl transferase in UK Biobank and HSE/SHeSs (**Table S6**). Schoenfeld residuals (**Figure S1**). Results of subgroup and sensitivity analyses for dose-response relationship between alcohol consumption and risk of all-cause mortality, cardiovascular mortality, and cardiovascular events (**Figure S2, S7-S12**). Patients inclusion flowchart for HSE/SHeSs and UK Biobank (**Figure S3**). Association of drinking categories with all-cause mortality, cardiovascular mortality, and cardiovascular events by cohort, sex, and primary events (**Figure S4-S5**). Study flow diagram (**Figure S6**). Funnel plots (**Figure S13**).

## Data Availability

Data from UK Biobank (http://www.ukbiobank.ac.uk/) and the Health Survey for England and the Scottish Health Survey (https://www.ukdataservice.ac.uk/) are available to researchers upon application.

## Abbreviations

AHA: American Heart Association
BMI: Body mass index
CI: Confidence interval
CVD: Cardiovascular disease
FP2: Second-degree fractional polynomial model
HES: Hospital Episode Statistics
HR: Hazard ratio
HSE: Health Survey for England
IQR: Interquartile range
MI: Myocardial infarction
RR: Relative risk
SHeSs: Scottish Health Survey
