# Supplementary material for: Association of alcohol consumption with morbidity and mortality in patients with cardiovascular disease: original data and meta-analysis of 48,423 men and women
Source: BMC Med. 2021 Jul 27;19:167. doi: 10.1186/s12916-021-02040-2 (PMC8314518; doi:10.1186/s12916-021-02040-2)
Supplement: Supplementary file 1 — Additional file 1: Supplementary Materials. Supplementary methods for de novo cohort analyses (Appendix S1 and Table S1). Quality assessment checklist (Appendix S2). Literature search strategy (Table S2). Alcohol consumption, effect estimates, and confounder adjustment reported by studies selected for meta-analyses (Table S3-S5). Associations of alcohol intake with HDL-cholesterol and gamma-glutamyl transferase in UK Biobank and HSE/SHeSs (Table S6). Schoenfeld residuals (Figure S1). Results of subgroup and sensitivity analyses for dose-response relationship between alcohol consumption and risk of all-cause mortality, cardiovascular mortality, and cardiovascular events (Figure S2, S7-S12). Patients inclusion flowchart for HSE/SHeSs and UK Biobank (Figure S3). Association of drinking categories with all-cause mortality, cardiovascular mortality, and cardiovascular events by cohort, sex, and primary events (Figure S4-S5). Study flow diagram (Figure S6). Funnel plots (Figure S13). [file 12916_2021_2040_MOESM1_ESM.docx]

SUPPLEMENTAL MATERIAL

[Appendix S1. Supplementary methods for *de novo* cohort analyses 1](#_Toc48828045)

[Table S1. ICD and OPCS codes used in analyses of UK Biobank and HSE/SHeSs 3](#_Toc48828046)

[Figure S1. Schoenfeld residuals 4](#_Toc48828047)

[Table S2. Literature search strategy 9](#_Toc48828048)

[Figure S2. Dose-response relationship between alcohol consumption and risk of all-cause mortality, cardiovascular mortality, and cardiovascular events. For open-ended upper categories, mean values were defined as lower boundary×1, lower boundary×1.4, and lower boundary×1.6 10](#_Toc48828049)

[Appendix S2. Quality assessment checklist 13](#_Toc48828050)

[Figure S3. Patients inclusion flowchart for HSE/SHeSs and UK Biobank 14](#_Toc48828051)

[Figure S4. Association of drinking categories with all-cause mortality, cardiovascular mortality, and cardiovascular events by cohort and sex 15](#_Toc48828052)

[Figure S5. Association of drinking categories with all-cause mortality, cardiovascular mortality, and cardiovascular events by cohort and primary cardiovascular events 17](#_Toc48828053)

[Figure S6. Study flow diagram 19](#_Toc48828054)

[Table S3. Alcohol consumption, effect estimates, and confounder adjustment reported by studies on all-cause mortality 20](#_Toc48828055)

[Table S4. Alcohol consumption, effect estimates, and confounder adjustment reported by studies on cardiovascular mortality 22](#_Toc48828056)

[Table S5. Alcohol consumption, effect estimates, and confounder adjustment reported by studies on cardiovascular events 24](#_Toc48828057)

[Figure S7. Dose-response relationship between alcohol consumption and risk of all-cause mortality, cardiovascular mortality, and cardiovascular events, stratified by sex 25](#_Toc48828058)

[Figure S8. Dose-response relationship between alcohol consumption and risk of all-cause mortality, cardiovascular mortality, and cardiovascular events, stratified by primary cardiovascular event 27](#_Toc48828059)

[Figure S9. Dose-response relationship between alcohol consumption and risk of all-cause mortality, cardiovascular mortality, and cardiovascular events, relative to different non-drinking reference group 30](#_Toc48828060)

[Figure S10. Dose-response relationship between alcohol consumption and risk of all-cause mortality, cardiovascular mortality and cardiovascular events, using different method of assessing alcohol consumption 32](#_Toc48828061)

[Figure S11. Overall dose-response relationship between alcohol consumption and risk of all-cause and cardiovascular mortality after excluding studies with a quality assessment score <7 34](#_Toc48828062)

[Figure S12. Overall dose-response relationship between alcohol consumption and risk of all-cause mortality, cardiovascular mortality, and cardiovascular events, using least adjusted estimates (adjusted for age, sex, and smoking status only) 35](#_Toc48828063)

[Figure S13. Funnel plots 36](#_Toc48828064)

[Table S6. Associations of alcohol intake with HDL-cholesterol and gamma-glutamyl transferase in UK Biobank and HSE/SHeSs 38](#_Toc48828065)

# Appendix S1. Supplementary methods for *de novo* cohort analyses

**Study cohorts**

Data were obtained from participants in the Health Survey for England (HSE) [65], the Scottish Health Survey (SHeSs) [66], and UK Biobank [67]. Complete cohort profiles are available via the above citations. Briefly, HSE/SHeSs is a series of surveys which use a multistage stratified design to draw a nationally representative sample of the general population living in England/Scottish households. Each survey year consists of a new sample of private residential addresses and participants and entails a household interview followed by a nurse visit to collect baseline information on demographics, anthropometry, self-reported health, and health-related behaviours. Participants have been asked for consent to follow-up through data linkage, thus converting cross-sectional survey data into a longitudinal study with samples from different survey years with a range of health outcomes. The present analyses combined data from the 1994–2008 HSE datasets and the 1995, 1998 and 2003 SHeSs datasets and were restricted to participants aged ≥16 years reporting to have been diagnosed with myocardial infarction (MI)/angina (not recorded separately) or stroke prior to baseline.

UK Biobank is a prospective study of more than 500000 participants, aged 40–69 years when recruited in 2006–2010. Participants were invited to attend one of 22 centres across England, Scotland, and Wales, where a touchscreen questionnaire was completed, a nurse-led interview was performed, and physical measurements were taken. We identified participants with MI, angina, or stroke before recruitment based on record linkage to the Hospital Episode Statistics (HES, 2 December 1980 onwards). Participants who had self-reported events at baseline assessment but without evidence from HES data were excluded from analyses. Algorithmic definitions developed by the UK Biobank Outcome Adjudication Group were applied for MI [68] and stroke [69]. We developed classification algorithms for angina using the process and data fields (diagnoses in the primary or any secondary position) recommended by the Group [70] with relevant codes from the International Classification of Diseases (ICD) Edition 9 and Edition 10 (Table S1) [71].

**Alcohol assessment**

At baseline of each cohort, participants were asked about their drinking status and were asked to report their average weekly or monthly consumption of different types of alcoholic beverages. These measures were then converted into standard UK units and summed to obtain an average alcohol consumption in units per week, where one unit contains 8g of ethanol [15] and is equivalent to half a pint of beer/lager/cider, half a glass of wine/champagne, one measure of spirits, or one glass of fortified wine [16]. Alcopops and other forms of alcohol count as 1.5 units [72]. We separated former drinkers from never drinkers and used never drinkers as the reference group to provide additional data for meta-analyses on different non-drinking reference group. Current drinkers were categorized into three groups in line with the UK guidelines: low-level drinkers (≤ 14 units per week), medium-level drinkers (>14 to ≤50 units per week for men, >14 to ≤35 units per week for women), and high-level drinkers (>50 units per week for men, >35 units per week for women) [17].

**Outcomes**

We assessed alcohol consumption in relation to three outcomes: all-cause mortality, cardiovascular mortality (ICD-10 codes I00–I99) [73] and major cardiovascular events (as defined below). Date and underlying cause of death (coded with ICD-10) were ascertained by national death registries and all cohorts contributed to the mortality analyses. We censored participants at their date of death, the date they left the UK or the end of follow-up (until 14 February 2011 in HSE, 31 December 2009 in SHeSs or 9 February 2018 in UK Biobank), whichever came first.

Cardiovascular events were a composite of angina, fatal and non-fatal MI and stroke, revascularization procedures (angioplasty or coronary artery bypass graft), death from heart failure, and sudden cardiac death, and only UK Biobank contributed data to this analysis. Non-fatal events were identified from linked HES records using primary diagnoses coded with ICD-10 and procedures coded with OPCS4 (the Office of Population Censuses and Surveys’ Classification of Interventions and Procedures Version 4), as given in Table S1. Any hospital or death records that occurred within 28 days of the date for a detected event were considered to relate to the same event [74]. Participants were followed up until the date of their first detected event or were censored on the date they left the UK or the last date of data linkage (31 March 2017).

**Covariates**

Covariates considered in analyses were assessed at baseline and included age, sex, smoking status (never, ex-, or current smoker), self-reported history of diabetes and hypertension, socioeconomic position/education, body mass index, and regular medications (cholesterol-lowering medications, antihypertensive medications, antiplatelet agents, digoxin, and warfarin). In HSE/SHeSs, socioeconomic position was defined using the participant’s occupational classification, categorised as low (semi-skilled or unskilled manual), intermediate (skilled non-manual or manual) or high (professional or managerial technical) [75]. For UK Biobank participants, highest educational qualification was used and categorised into four levels: None; O levels/GCSEs, CSEs or equivalent; A/AS levels, NVQ or HND or HNC or equivalent, or other professional qualification; College or university degree [76].

# Table S1. ICD and OPCS codes used in analyses of UK Biobank and HSE/SHeSs

|  | | |
| --- | --- | --- |
| Angina ^[71]^ | | |
| ICD-9 | 411, 4119, 413, 4139 | |
| ICD-10 | I20, I20.0, I20.1, I20.8, I20.9 | |
| MI ^[68]^ | | |
| ICD-10 | MI, unclassified | I21, I22, I23, I23.0, I23.1, I23.2, I23.3, I23.4, I23.5, I23.6, I23.8, I24.1, I25.2 |
| ICD-10 | ST elevation MI | I21.0, I21.1, I21.2, I21.3, I22.0, I22.1, I22.8 |
| ICD-10 | Non-ST elevation MI | I21.4, I21.9, I22.9 |
| Stroke ^[69]^ | | |
| ICD-10 | Ischaemic stroke | I63, I63.0, I63.1, I63.2, I63.3, I63.4, I63.5, I63.6, I63.8, I63.9, I64.X |
| ICD-10 | Intracerebral haemorrhage | I61, I61.0, I61.1, I61.2, I61.3, I61.4, I61.5, I61.6, I61.8, I61.9 |
| ICD-10 | Subarachnoid haemorrhage | I60, I60.0, I60.1, I60.2, I60.3, I60.4, I60.5, I60.6, I60.7, I60.8, I60.9 |
| Heart failure ^[77]^ | | |
| ICD-10 | I11.0, I13.0, I13.2, I25.5, I42.0, I42.5, I42.8, I42.9, I50.0, I50.1, I50.9 | |
| Sudden death ^[78]^ | | |
| ICD-10 | I46.1, I49.9, R96, R96.0, R96.1 | |
| Revascularization procedures ^[79]^ | | |
| OPCS4 | Coronary artery bypass graft | K40, K41, K42, K43, K44, K45, K46 |
| OPCS4 | Percutaneous transluminal coronary angioplasty | K49, K50, K75 |
| ICD=the International Classification of Diseases, MI=myocardial infarction, OPCS=OPCS Classification of Interventions and Procedures | | |

# Figure S1. Schoenfeld residuals

a. All-cause mortality for Health Survey for England/Scottish Health Survey models


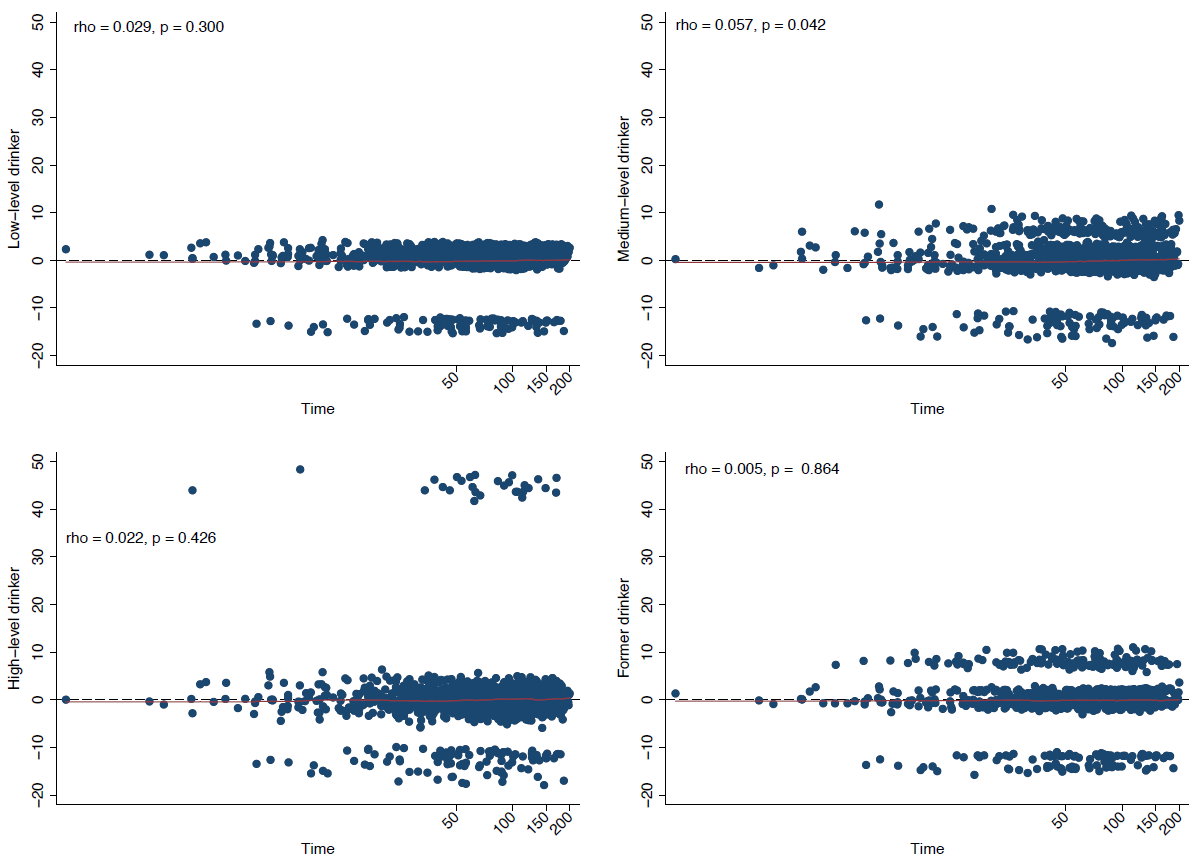


b. Cardiovascular mortality for Health Survey for England/Scottish Health Survey models


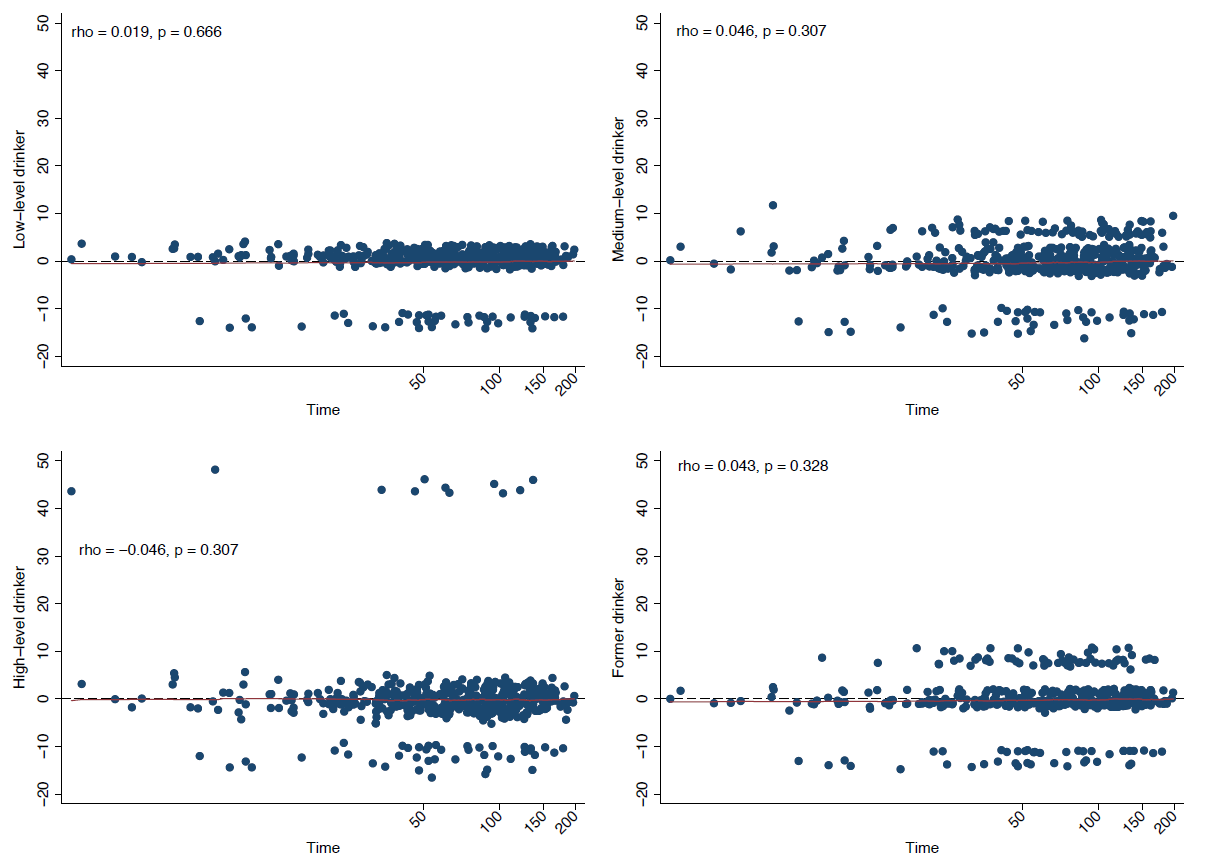


c. All-cause mortality for UK Biobank models


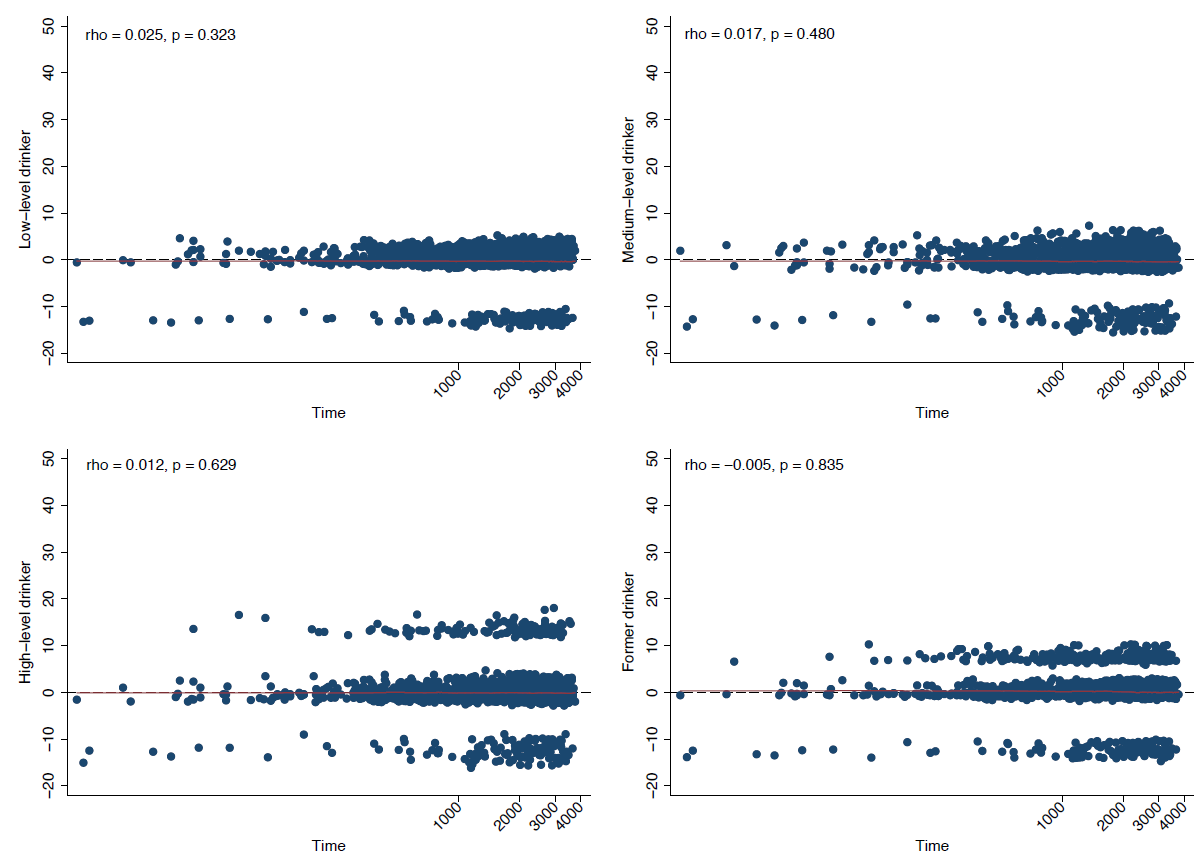


d. Cardiovascular mortality for UK Biobank models

**
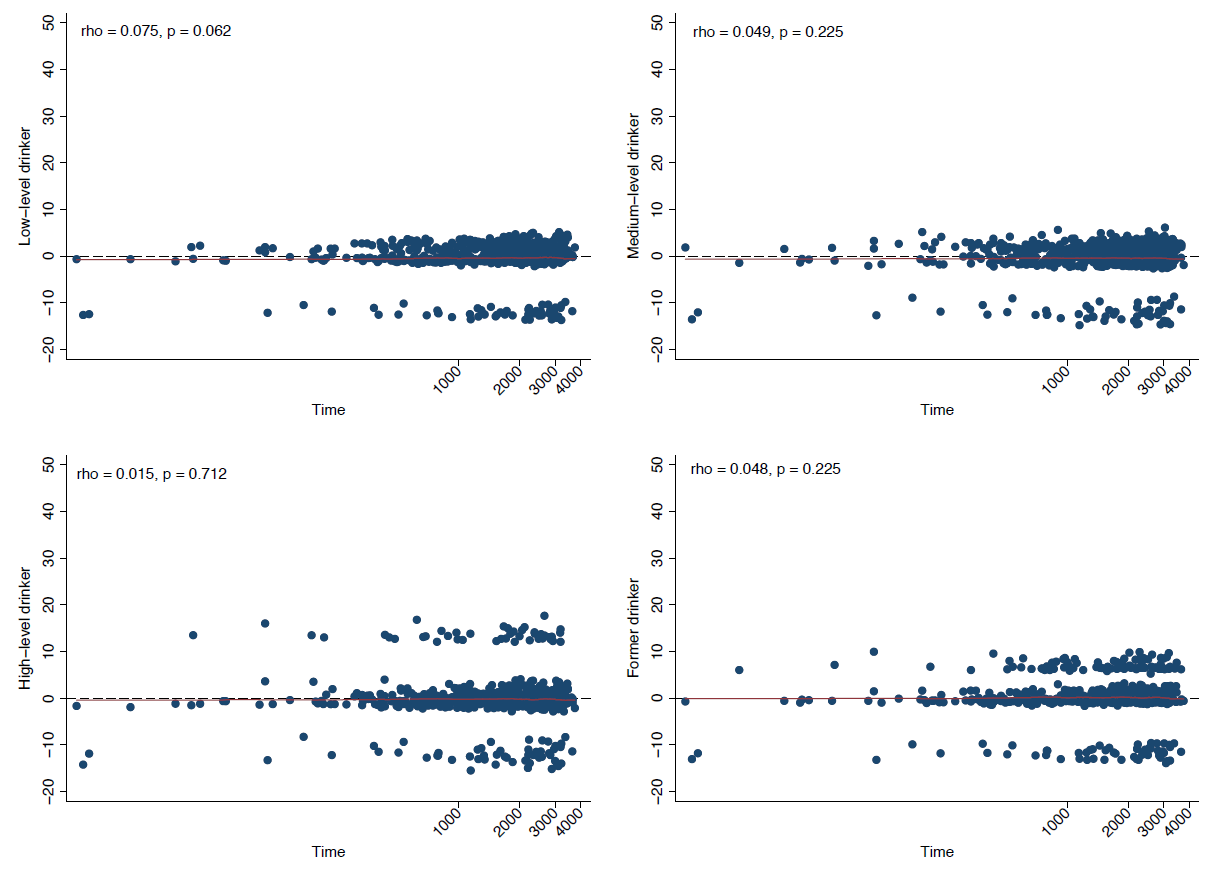
**

e. Cardiovascular events for UK Biobank models

**
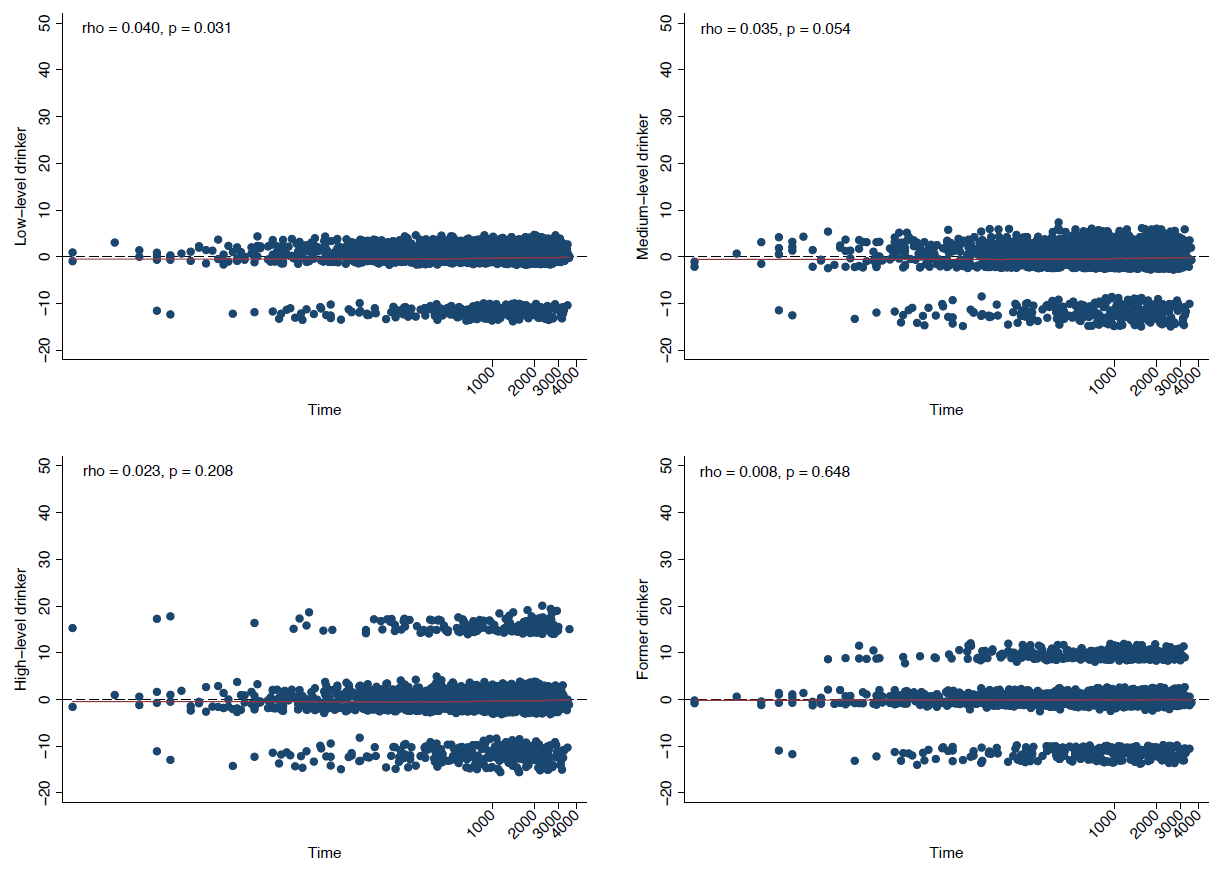
**

# Table S2. Literature search strategy

|  | | |
| --- | --- | --- |
| **#** | **Medline (Ovid)** | **Results** |
| 1 | Alcohol Drinking/ | 66993 |
| 2 | ((alcohol or beer$1 or wine$1 or spirit or spirits or liquor$1 or liqueur$1) adj2 (intake$1 or consum$ or drink$)).ab,ti. | 68862 |
| 3 | exp myocardial infarction/ or exp coronary disease/ | 363351 |
| 4 | ((isch?emic heart disease$1 or IHD or myocardial isch?emia or myocardial infarct$ or MI or acute myocardial infarct$ or MI or coronary disease$1 or coronary artery disease$1 or CAD or coronary heart disease$1 or CHD or heart disease$1 or cardiovascular disease$1 or CVD or angina) adj2 (patients or people or women or men)).ab,ti. | 78580 |
| 5 | ((myocardial infarct$ or MI or acute myocardial infarct$ or MI) adj2 (surviv$ or after or following)).ab,ti. | 31387 |
| 6 | exp STROKE/ | 134621 |
| 7 | ((stroke or strokes or acute cerebrovascular accident$1 or cerebrovascular accident$1 or CVA$1 or apoplexy or brain vascular accident$1) adj2 (patients or people or women or men or surviv$ or after or following)).ab,ti. | 67330 |
| 8 | exp cohort studies/ or exp follow-up studies/ or longitudinal studies/ | 2014690 |
| 9 | (comment or editorial or letter or case reports or news or review or meta analysis).pt. | 6532171 |
| 10 | 1 or 2 | 106345 |
| 11 | 3 or 4 or 5 or 6 or 7 | 551960 |
| 12 | 8 and 10 and 11 | 1128 |
| 13 | limit 12 to humans | 1128 |
| 14 | 13 not 9 | 1070 |
| **#** | **Embase (Ovid)** | **Results** |
| 1 | exp drinking behavior/ | 47562 |
| 2 | ((alcohol or beer$1 or wine$1 or spirit or spirits or liquor$1 or liqueur$1) adj2 (intake$1 or consum$ or drink$)).ab,ti. | 94229 |
| 3 | exp heart infarction/ or exp coronary artery disease/ | 593221 |
| 4 | ((isch?emic heart disease$1 or IHD or myocardial isch?emia or myocardial infarct$ or MI or acute myocardial infarct$ or MI or coronary disease$1 or coronary artery disease$1 or CAD or coronary heart disease$1 or CHD or heart disease$1 or cardiovascular disease$1 or CVD or angina) adj2 (patients or people or women or men)).ab,ti. | 115149 |
| 5 | ((myocardial infarct$ or MI or acute myocardial infarct$ or MI) adj2 (surviv$ or after or following)).ab,ti. | 42351 |
| 6 | exp cerebrovascular accident/ | 209214 |
| 7 | ((stroke or strokes or acute cerebrovascular accident$1 or cerebrovascular accident$1 or CVA$1 or apoplexy or brain vascular accident$1) adj2 (patients or people or women or men or surviv$ or after or following)).ab,ti. | 111595 |
| 8 | exp follow up/ or longitudinal study/ | 1663997 |
| 9 | (Patent or Tombstone or Note or Editorial or Letter or Erratum or Books or Chapter or Review).pt. | 5351012 |
| 10 | 1 or 2 | 122144 |
| 11 | 3 or 4 or 5 or 6 or 7 | 859770 |
| 12 | 8 and 10 and 11 | 1039 |
| 13 | limit 12 to human | 996 |
| 14 | 13 not 9 | 960 |

# Figure S2. Dose-response relationship between alcohol consumption and risk of all-cause mortality, cardiovascular mortality, and cardiovascular events. For open-ended upper categories, mean values were defined as lower boundary×1, lower boundary×1.4, and lower boundary×1.6

Best-fitting second-degree fractional polynomial models (with 95% CIs) are shown in solid curves with each data point overlaid as circles. Circle size indicates the weighting of each data point and is inversely proportional to the variance of the log-transformed relative risk.

a. All-cause mortality

***
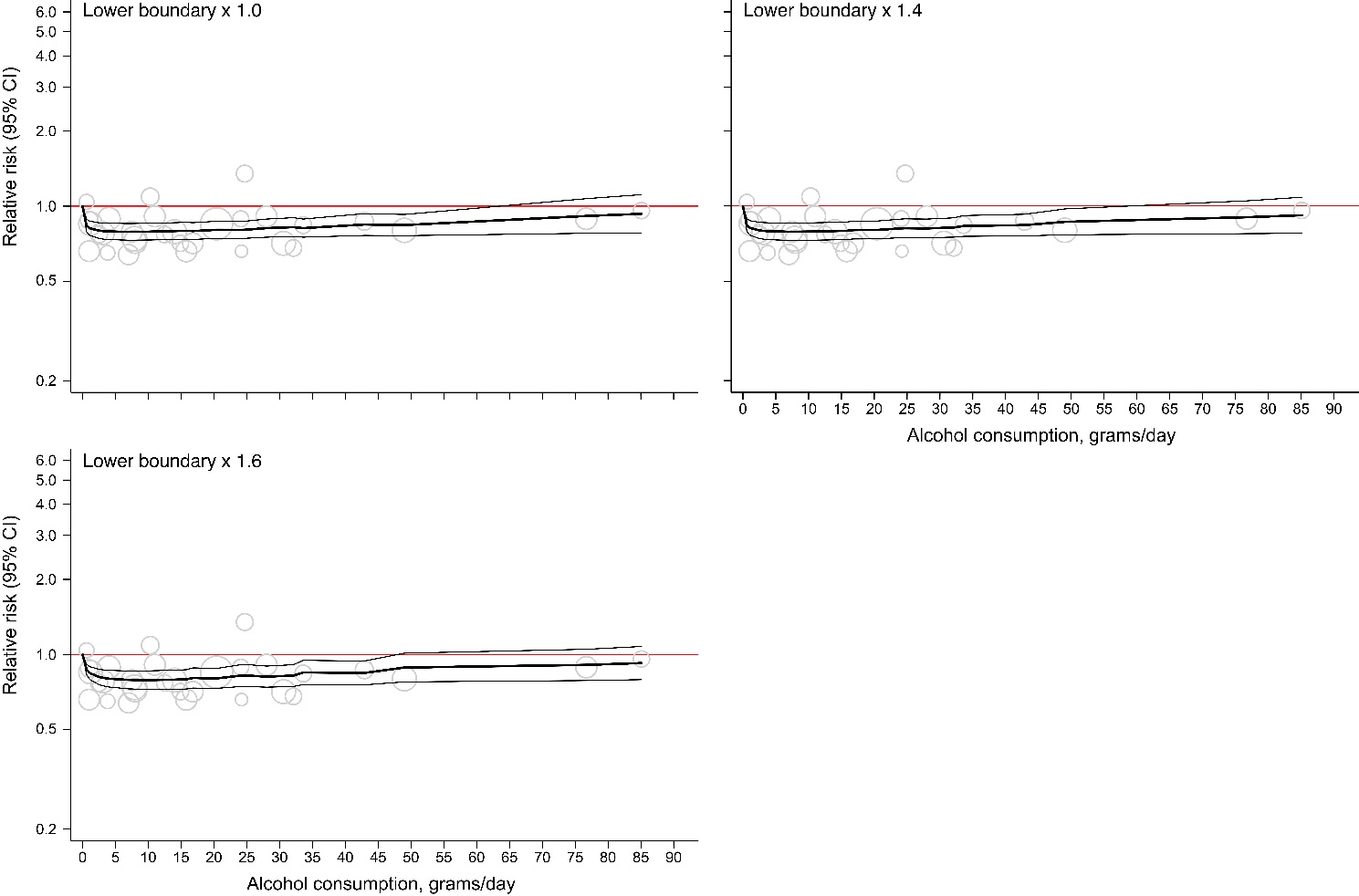
***

b. Cardiovascular mortality

***
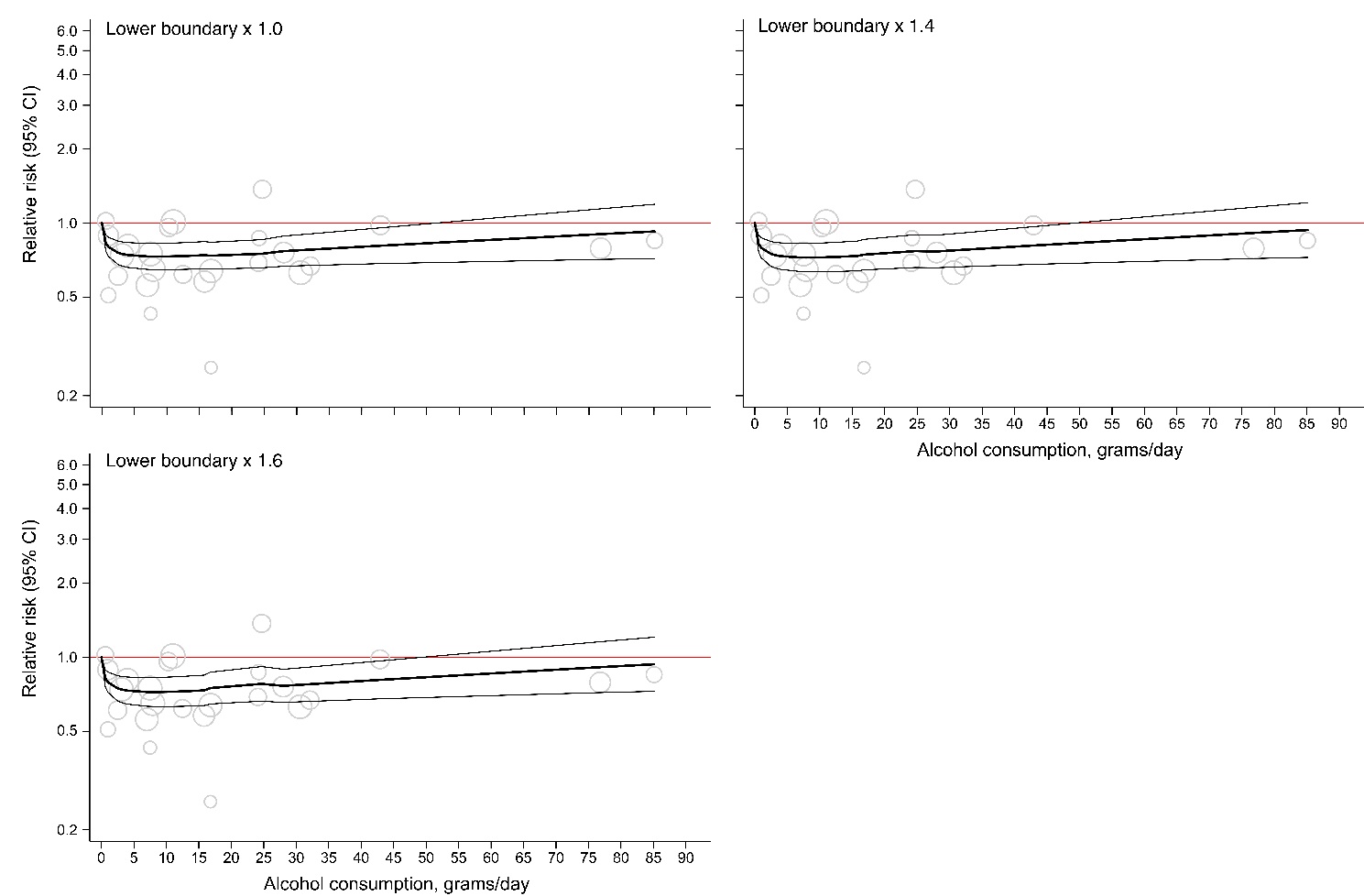
***

c. Cardiovascular events

***
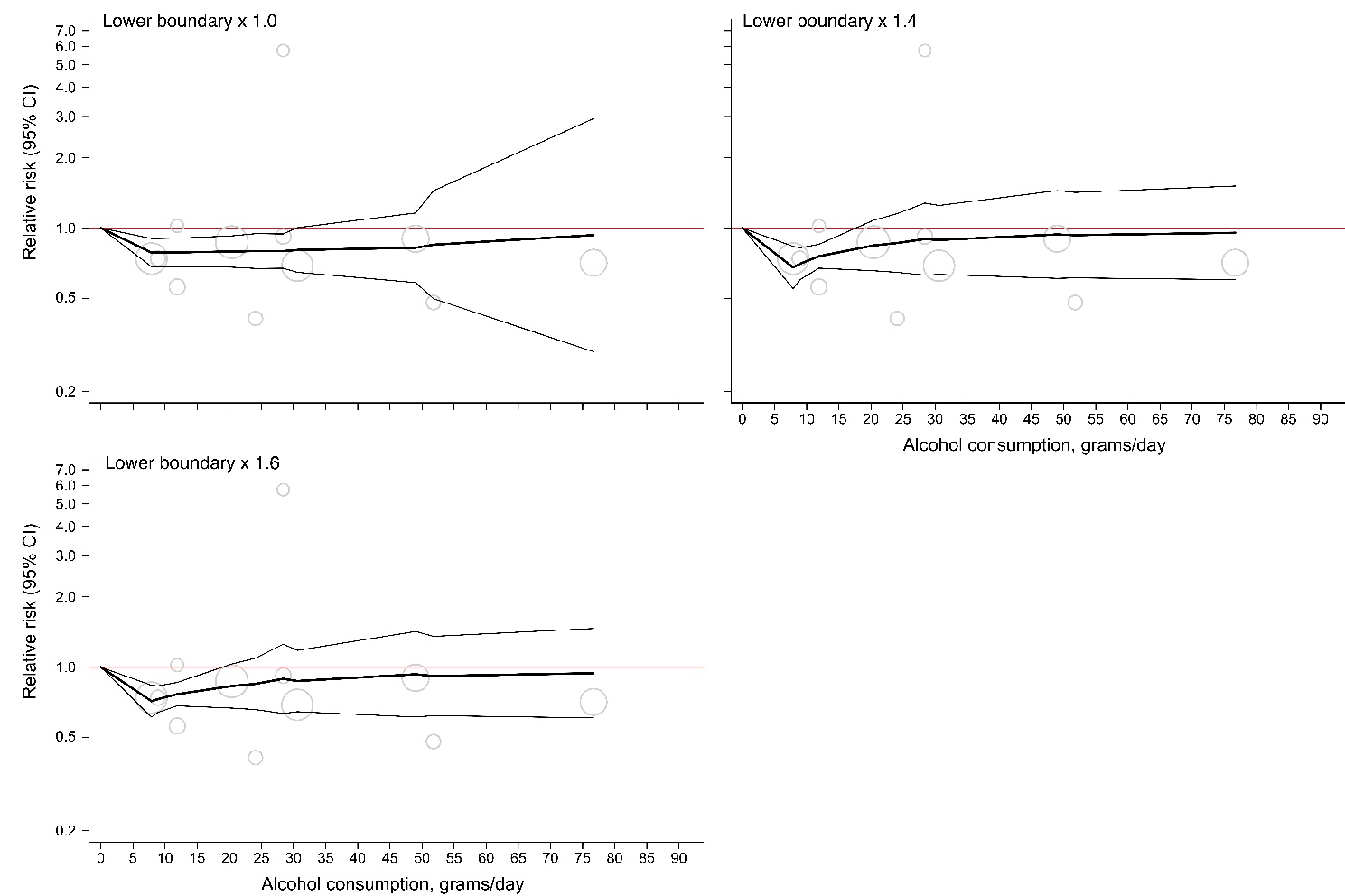
***

# Appendix S2. Quality assessment checklist

**NEWCASTLE - OTTAWA QUALITY ASSESSMENT SCALE
COHORT STUDIES**

Note: A study can be awarded a maximum of one star for each numbered item within the Selection and Outcome categories. A maximum of two stars can be given for Comparability.

**Selection**

1) Representativeness of the exposed cohort
a) truly representative of the average *current drinkers* in the community *****b) somewhat representative of the average *current drinkers* in the community *****c) selected group of users (e.g. nurses, volunteers)
d) no description of the derivation of the cohort

2) Selection of the non-exposed cohort
a) drawn from the same community as the exposed cohort *****b) drawn from a different source
c) no description of the derivation of the non-exposed cohort

3) Ascertainment of exposure
a) secure record (e.g. surgical records) *****b) structured interview *****c) written self-report
d) no description

4) Demonstration that outcome of interest was not present at start of study
a) yes *****b) no

**Comparability**

1) Comparability of cohorts on the basis of the design or analysis
a) study controls for *smoking status* *****b) study controls for any additional factor *****

**Outcome**

1) Assessment of outcome
a) independent blind assessment *****b) record linkage *****c) self-report
d) no description

2) Was follow-up long enough for outcomes to occur
a) yes, *at least six years duration* *****b) no

3) Adequacy of follow up of cohorts
a) complete follow up: all subjects accounted for *****b) subjects lost to follow up unlikely to introduce bias:
 small number lost (> *95*% follow up) or description provided of those lost *****c) follow up rate < *95*% and no description of those lost
d) no statement

***Cohort samples at initial exposure assessment***

HSE/SHeSs=38012, UK Biobank=502536

***Participants with pre-existing angina, MI or stroke***

HSE/SHeSs participants with self-reported MI/angina or stroke (n=6270)

UK Biobank participants with HES confirmed angina, MI, or stroke (n=18532)

***Participants excluded, with reasons***

HSE/SHeSs:

- Alcohol measurements unavailable (n=1900)
- Missing covariates (n=1564)
- Follow-up data unavailable (n=4)

UK Biobank:

- Drinking status unknown (n=91)
- Alcohol measurements unavailable (n=3333)
- Missing covariates (n=722)

***Participants included in analyses***

HSE/SHeSs (n=2802):

- MI/angina patients=2341, stroke=535

UK Biobank (n=14386):

- MI patients=5333, angina=9589, stroke=2064

# Figure S3. Patients inclusion flowchart for HSE/SHeSs and UK Biobank

HES=[hospital episode statistics,](https://biobank.ndph.ox.ac.uk/showcase/refer.cgi?id=138483) HSE=the Health Survey for England, MI= myocardial infarction, SHeSs=the Scottish Health Survey

# Figure S4. Association of drinking categories with all-cause mortality, cardiovascular mortality, and cardiovascular events by cohort and sex

All models were adjusted for age, smoking status, diabetes, hypertension, socioeconomic position or education, body mass index, cholesterol-lowering medications, antihypertensive medications, antiplatelet agents, digoxin, and warfarin.

CI=confidence interval, HR=hazard ratio, HSE=the Health Survey for England, SHeSs= the Scottish Health Survey

a. All-cause mortality


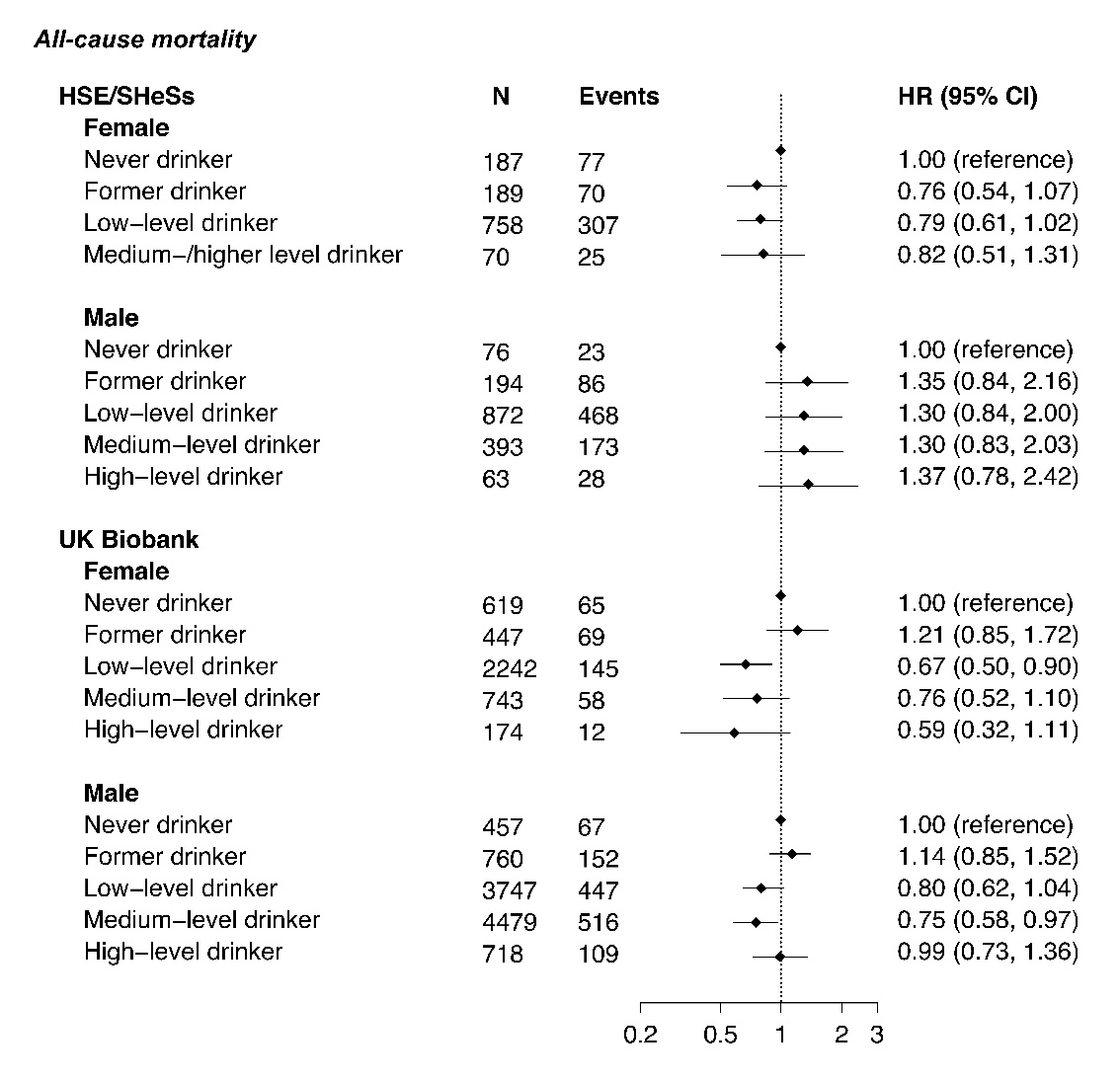


b. Cardiovascular mortality


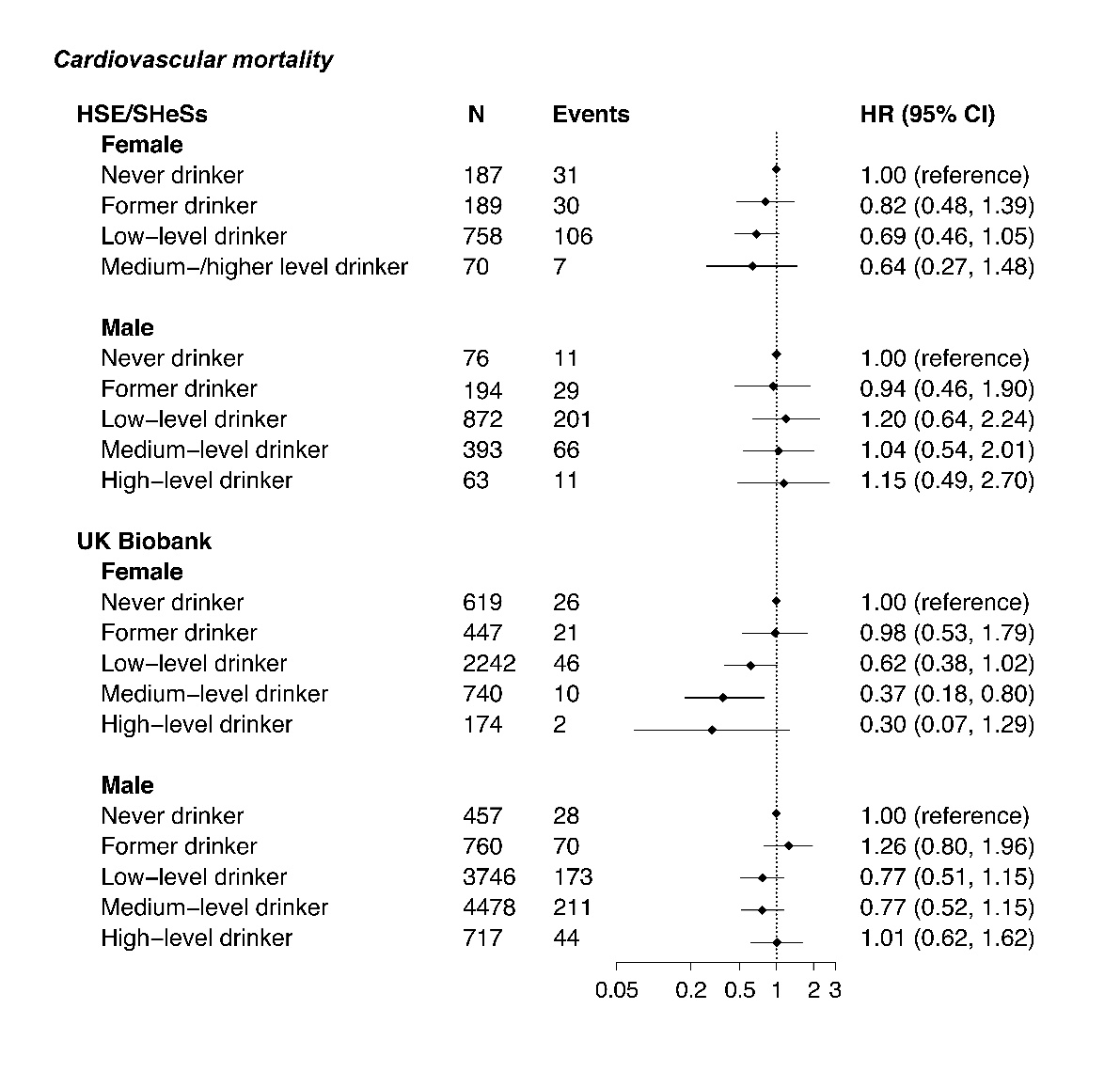


c. Cardiovascular events


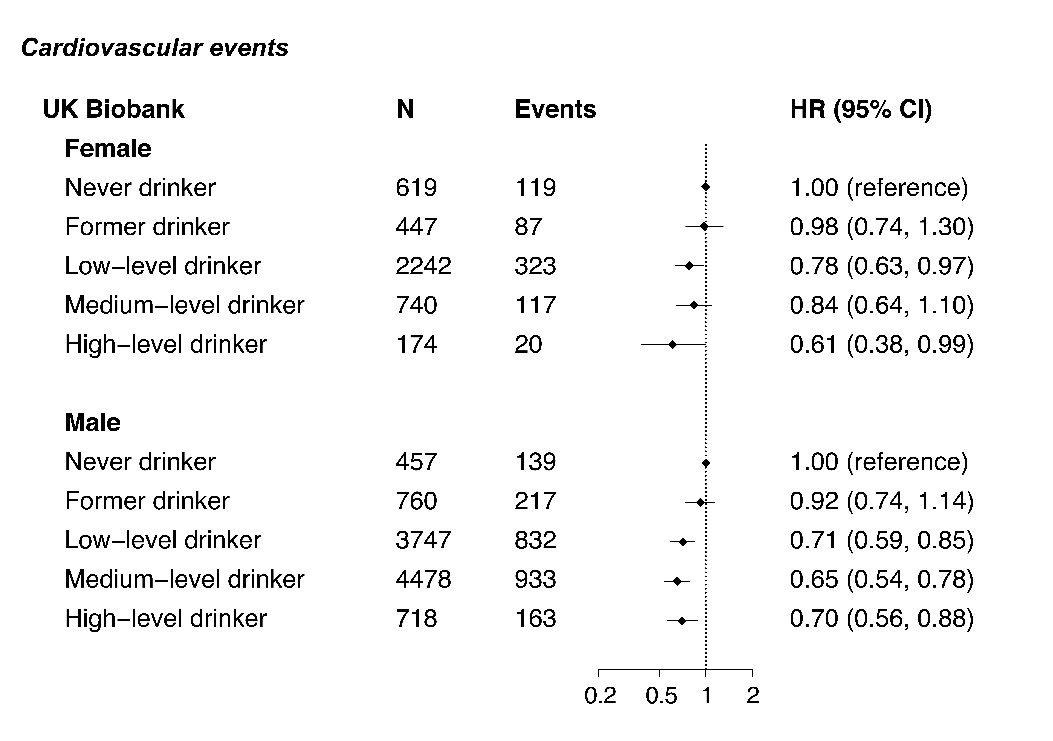


# Figure S5. Association of drinking categories with all-cause mortality, cardiovascular mortality, and cardiovascular events by cohort and primary cardiovascular events

Models for MI, angina, and stroke as primary event were adjusted for each other as well as age, sex, smoking status, diabetes, hypertension, socioeconomic position or education, body mass index, cholesterol-lowering medications, antihypertensive medications, antiplatelet agents, digoxin, and warfarin.

CI=confidence interval, HR=hazard ratio, HSE=the Health Survey for England, MI= myocardial infarction, SHeSs= the Scottish Health Survey

a. All-cause mortality


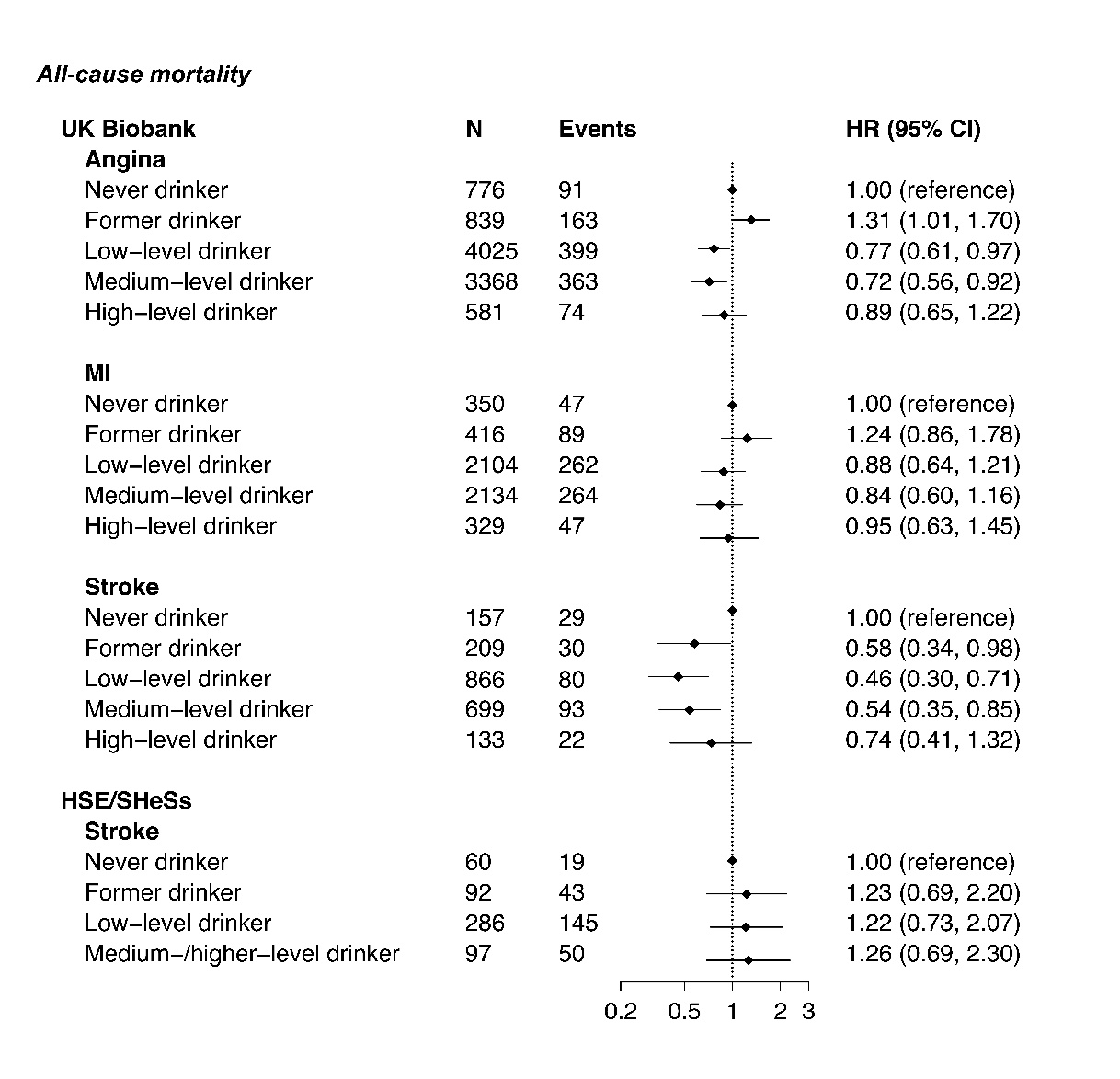


b. Cardiovascular mortality


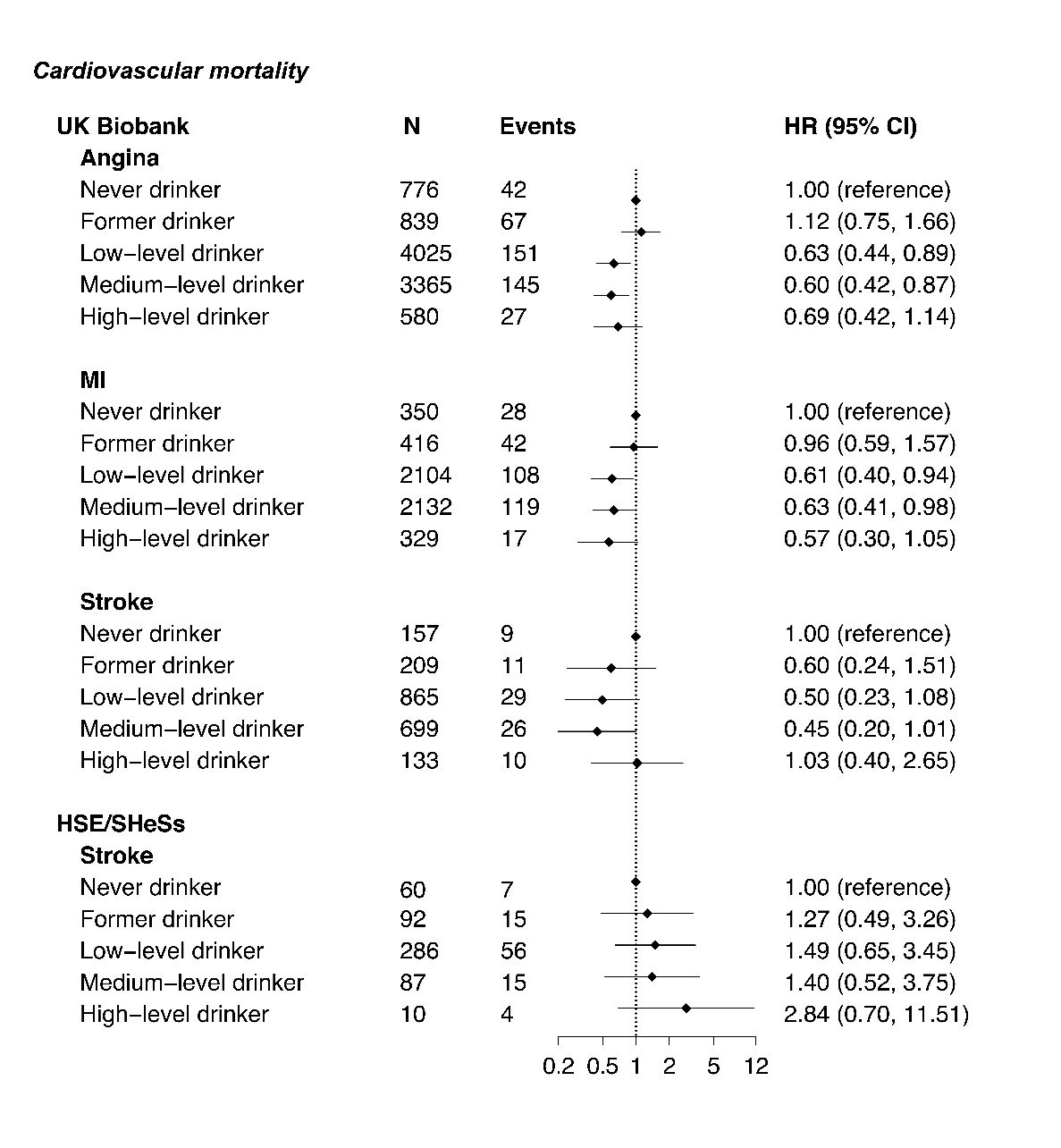


c. Cardiovascular events


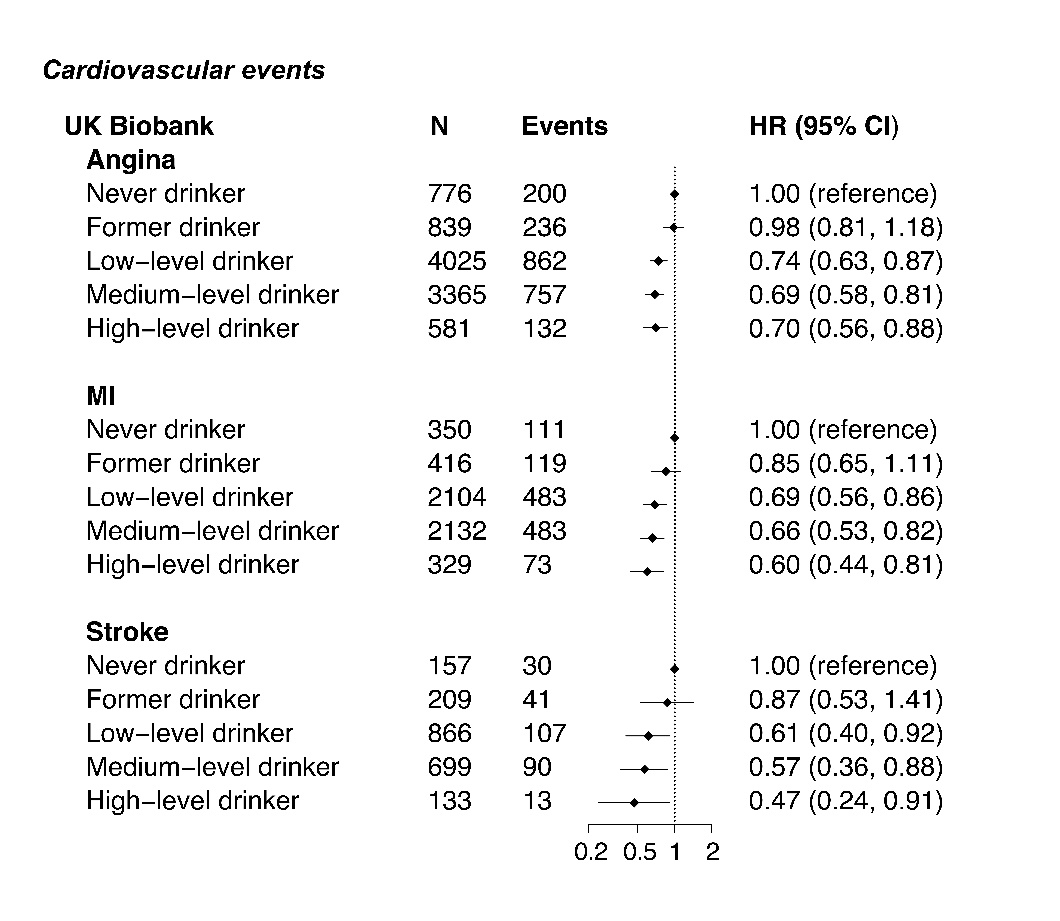


Records identified through database searching: Medline=1070, Embase=960

Additional records identified by checking reference lists (n=3)

Identification

Included

Eligibility

Screening

Full-text articles excluded, with reasons:

- Outcomes other than ACM, CVM, or CVE (n=27)
- Only two categories of alcohol intake (drinkers versus non-drinkers, n=10)
- No risk estimates or frequency counts (n=4)
- Multiple reports from the same dataset (n=1)

Records after duplicates removed (n=1722)

Data extraction and eligibility verification (n=54)

Full-text articles excluded, with reasons:

- Study population not CVD patients (n=17)
- Not longitudinal study design (n=9)
- Alcohol intake not as the main exposure (n=11)
- Multiple reports from the same dataset (n=3)
- Conference abstract (n=15)

Records excluded, with reasons:

- Non-primary study (n=43)
- Study population not CVD patients (n=1285)
- Not longitudinal study design (n=122)
- Alcohol intake not as the main exposure (n=155)
- Multiple reports from the same dataset (n=8)

Full-text articles assessed for eligibility (n=109)

Published studies included in meta-analysis (n=12)

# Figure S6. Study flow diagram

ACM=all-cause mortality, CVD=cardiovascular disease, CVE=cardiovascular events, CVM=cardiovascular mortality

# Table S3. Alcohol consumption, effect estimates, and confounder adjustment reported by studies on all-cause mortality

| **First author, year** | **Alcohol consumption** | |  | **Risk of all-cause mortality** | | | | |
| --- | --- | --- | --- | --- | --- | --- | --- | --- |
|  | **Reported exposure categories** | **Estimated g/day *†** |  | **Total (N)** | **Cases (n)** | **Measure of association** | **Effect estimates** | **Confounder adjustment** |
| Levantesi, 2013 | Never/almost never | 0.0 |  | 3713 | 645 | HR | 1.00 (reference) | Age, gender, BMI, smoking, prior MI, history of hypertension, DM, peripheral vascular disease, electrical instability, exercise, LVEF, NYHA class, revascularization procedures, intakes of cooked vegetables, raw vegetables, fruit, fish, olive oil, other oil, butter, cheese, and coffee, use of n−3 PUFA, vitamin-E, antiplatelet agents, angiotensin-converting-enzyme inhibitor, lipid-lowering medication , beta-blockers |
|  | ≤0.5 L/day | 20.4 |  | 5821 | 874 |  | 0.85 (0.76–0.95) |  |
|  | >0.5 L/day | 49.0 |  | 985 | 137 |  | 0.80 (0.66–0.98) |  |
| Pai, 2012 | 0 g/day | 0.0 |  | 515 | 168 | HR | 1.00 (reference) | Age at diagnosis, questionnaire follow-up cycle, smoking, BMI, physical activity, diabetes, hypertension, lipid-lowering medication, aspirin use, heart failure at MI |
|  | 0.1-9.9 g/day | 3.1 |  | 719 | 161 |  | 0.78 (0.62–0.97) |  |
|  | 10.0-29.9 g/day | 15.8 |  | 420 | 97 |  | 0.66 (0.51–0.86) |  |
|  | ≥30.0 g/day | 42.9 |  | 164 | 42 |  | 0.87 (0.61–1.25) |  |
| Rosenbloom, 2012 | None | 0.0 |  | 761 | 331 | HR | 1.00 (reference) | Age, BMI, previous MI, congestive HF, angina, DM, hypertension, non-cardiac co-morbidity, previous medication use, smoking, physical activity, income, education, marital status, race, peak creatine kinase level, receipt of thrombolytic therapy, congestive HF and ventricular tachycardia during hospitalization |
|  | <1 serving/week | 1.0 |  | 280 | 70 |  | 0.66 (0.50–0.86) |  |
|  | ≥1 to <3 servings/week | 3.8 |  | 75 | 15 |  | 0.65 (0.38–1.11) |  |
|  | ≥3 servings/week | 14.9 |  | 137 | 25 |  | 0.71 (0.46–1.09) |  |
| Janszky, 2008 | Longer-term abstainers | 0.0 |  | 140 | 35 | HR | 1.00 (reference) | Age, sex, smoking, obesity, self-reported physical activity, history of DM, education |
|  | >0 to <5 g/day | 2.5 |  | 437 | 84 |  | 0.77 (0.51–1.15) |  |
|  | 5–20 g/day | 12.5 |  | 447 | 80 |  | 0.77 (0.50–1.18) |  |
|  | over 20 g/day | 24.1 |  | 308 | 60 |  | 0.89 (0.56–1.40) |  |
| Aguilar, 2004 | 0 drink/week | 0.0 |  | 1437 | 274 | HR | 1.00 (reference) | Age, gender, LVEF, prior MI, history of hypertension, history of DM, BMI, tobacco use, New York Heart Association classification, Killip class, beta-blocker use at the time of randomization, thrombolytic therapy with the qualifying MI, treatment (captopril) assignment |
|  | 1 to 10 drinks/week | 11 |  | 532 | 74 |  | 0.91 (0.70–1.19) |  |
|  | >10 drinks/week | 24.2 |  | 67 | 7 |  | 0.66 (0.31–1.41) |  |
| Jackson, 2003 | Rarely/never | 0.0 |  | 361 | 128 | RR | 1.00 (reference) | Age, smoking, diabetes mellitus, body mass index, exercise, angina, MI |
|  | <1 drink/week | 1.0 |  | 133 | 39 |  | 0.88 (0.60-1.28) |  |
|  | 1–6 drinks/week | 7.0 |  | 417 | 93 |  | 0.64 (0.48-0.85) |  |
|  | ≥1 drink/day | 16.8 |  | 409 | 109 |  | 0.71 (0.54-0.94) |  |
|  |  |  |  |  |  |  |  | ***(continued)*** |
| **First author, year** | **Alcohol consumption** | |  | **Risk of all-cause mortality** | | | | |
|  | **Reported exposure categories** | **Estimated g/day *†** |  | **Total (N)** | **Cases (n)** | **Measure of association** | **Effect estimates** | **Confounder adjustment** |
| Mukamal, 2001 | Abstainers | 0.0 |  | 896 | 196 | HR | 1.00 (reference) | Age, sex, use of thrombolytic therapy, peak creatine kinase level, congestive heart failure during index hospitalization, ventricular tachycardia during index hospitalization, and propensity score |
|  | <7 drinks/week | 7.5 |  | 696 | 91 |  | 0.79 (0.60-1.03) |  |
|  | ≥7 drinks/week | 32.1 |  | 321 | 30 |  | 0.68 (0.45-1.05) |  |
| Shaper, 2000 | Teetotallers | 0.0 |  | 43 | 18 | RR ‡ | 0.96 (0.57–1.62) | Age, smoking, social class, BMI, pre-existing diabetes, stroke, and regular medication |
|  | < 1 unit/week | 0.6 |  | 199 | 85 |  | 1.00 (reference) |  |
|  | 1–15 units/week | 10.3 |  | 230 | 94 |  | 1.05 (0.78–1.42) |  |
|  | > 16 units/week | 24.7 |  | 124 | 61 |  | 1.30 (0.93–1.83) |  |
| Muntwyler, 1998 | Rarely/never | 0.0 |  | 1125 | 240 | RR | 1.00 (reference) | Age, smoking, diabetes, physical activity, BMI |
|  | 1–4 drinks/month | 1.2 |  | 1227 | 211 |  | 0.85 (0.69–1.05) |  |
|  | 2–6 drinks/week | 8.0 |  | 1390 | 187 |  | 0.72 (0.58–0.89) |  |
|  | 1 drinks/day | 14.0 |  | 1424 | 249 |  | 0.79 (0.64–0.96) |  |
|  | ≥2 drinks/day | 33.6 |  | 192 | 33 |  | 0.84 (0.55–1.26) |  |
| HSE/SHeSs § | Never drinker | 0.0 |  | 263 | 100 | HR | 1.00 (reference) | Age, sex, smoking, socioeconomic position, history of DM, hypertension, BMI, cholesterol-lowering medications, antihypertensive medications, antiplatelet agents, digoxin |
|  | Low-level drinker | 4.0 |  | 1630 | 775 |  | 0.89 (0.71–1.11) |  |
|  | Medium-level drinker | 28.0 |  | 458 | 198 |  | 0.91 (0.70–1.18) |  |
|  | High-level drinker | 85.1 |  | 68 | 28 |  | 0.96 (0.62–1.49) |  |
| UK Biobank § | Never drinker | 0.0 |  | 1076 | 132 | HR | 1.00 (reference) | Age, sex, smoking, education, history of DM, hypertension, BMI, cholesterol-lowering medications, antihypertensive medications, antiplatelet agents, digoxin, warfarin |
|  | Low-level drinker | 7.9 |  | 5989 | 592 |  | 0.74 (0.61–0.89) |  |
|  | Medium-level drinker | 30.6 |  | 5222 | 574 |  | 0.71 (0.58–0.87) |  |
|  | High-level drinker | 76.7 |  | 892 | 121 |  | 0.89 (0.69–1.15) |  |
| * The upper limit of the highest exposure category defined as the lower bound multiplied by 1.2, unless explicitly defined within each publication  † Average intake in each consumption category. Where unreported, the median of the upper and lower bounds was used  ‡ Effect estimates re-calculated according to a reference group other than that originally reported. This was undertaken using the Hamling method, as described in text  § Measures of usual weekly consumption are presented in line with the current UK guidelines, categorized as never drinker, low-level drinker (≤ 14 units/week), medium-level drinker ( >14 to ≤50 units/week for men, >14 to ≤35 units/week for women), or high-level drinker (>50 units/week for men, >35 units/week for women) | | | | | | | | |

# Table S4. Alcohol consumption, effect estimates, and confounder adjustment reported by studies on cardiovascular mortality

| **First author, year** | **Alcohol consumption** | |  | **Risk of cardiovascular mortality** | | | | |
| --- | --- | --- | --- | --- | --- | --- | --- | --- |
|  | **Reported exposure categories** | **Estimated g/day *†** |  | **Total (N)** | **Cases (n)** | **Measure of association** | **Effect estimates** | **Confounder adjustment** |
| Pai, 2012 | 0 g/day | 0.0 |  | 515 | 92 | HR | 1.00 (reference) | Age at diagnosis, questionnaire follow-up cycle, smoking, BMI, physical activity, diabetes, hypertension, lipid-lowering medication, aspirin use, heart failure at MI |
|  | 0.1-9.9 g/day | 3.1 |  | 719 | 81 |  | 0.74 (0.54–1.02) |  |
|  | 10.0-29.9 g/day | 15.8 |  | 420 | 47 |  | 0.58 (0.39–0.84) |  |
|  | ≥30.0 g/day | 42.9 |  | 164 | 23 |  | 0.98 (0.60–1.60) |  |
| Janszky, 2008 | Longer-term abstainers | 0 |  | 140 | 23 | HR | 1.00 (reference) | Age, sex, smoking, obesity, self-reported physical activity, history of DM, education |
|  | >0 to <5 g | 2.5 |  | 437 | 44 |  | 0.61 (0.36–1.02) |  |
|  | 5–20 g | 12.5 |  | 447 | 42 |  | 0.62 (0.36–1.07) |  |
|  | over 20 g | 24.1 |  | 308 | 31 |  | 0.69 (0.38–1.25) |  |
| Aguilar, 2004 | 0 drink/week | 0 |  | 1437 | 215 | HR | 1.00 (reference) | Age, gender, LVEF, prior MI, history of hypertension, history of DM, BMI, tobacco use, New York Heart Association classification, Killip class, beta-blocker use at the time of randomization, thrombolytic therapy with the qualifying MI, treatment (captopril) assignment |
|  | 1 to 10 drinks/week | 11 |  | 532 | 62 |  | 1.00 (0.75–1.34) |  |
|  | >10 drinks/week | 24.2 |  | 67 | 7 |  | 0.87 (0.40–1.87) |  |
| Jackson, 2003 | Rarely/never | 0.0 |  | 361 | 101 | RR | 1.00 (reference) | Age, smoking, diabetes mellitus, body mass index, exercise, angina, MI |
|  | <1 drink/week | 1.0 |  | 133 | 29 |  | 0.89 (0.58-1.36) |  |
|  | 1–6 drinks/week | 7.0 |  | 417 | 62 |  | 0.56 (0.40-0.79) |  |
|  | ≥1 drink/day | 16.8 |  | 409 | 75 |  | 0.64 (0.46-0.88) |  |
| Mukamal, 2001 | Abstainers | 0 |  | 896 | 153 | HR | 1.00 (reference) | Age, sex, use of thrombolytic therapy, peak creatine kinase level, congestive heart failure during index hospitalization, ventricular tachycardia during index hospitalization, and propensity score |
|  | <7 drinks/week | 7.5 |  | 696 | 64 |  | 0.75 (0.55-1.02) |  |
|  | ≥7 drinks/week | 32.1 |  | 321 | 21 |  | 0.67 (0.41-1.17) |  |
| Shaper, 2000 | Teetotallers | 0 |  | 43 | 13 | RR ‡ | 0.98 (0.53–1.82) | Age, smoking, social class, BMI, pre-existing diabetes, stroke, and regular medication |
|  | < 1 unit/week | 0.6 |  | 199 | 62 |  | 1.00 (reference) |  |
|  | 1–15 units/week | 10.3 |  | 230 | 62 |  | 0.94 (0.65–1.35) |  |
|  | > 16 units/week | 24.7 |  | 124 | 47 |  | 1.34 (0.91–1.98) |  |
| Valmadrid, 1999 | Never drinkers | 0 |  | 31 | 12 | RR | 1.00 (reference) | Age, sex, cigarette smoking, insulin use, glycosylated hemoglobin level, plasma C-peptide level, digoxin use, the presence and severity of diabetic retinopathy |
|  | <2 g/day | 1 |  | 87 | 27 |  | 0.51 (0.24-1.12) |  |
|  | 2-13 g/day | 7.5 |  | 20 | 8 |  | 0.43 (0.15-1.22) |  |
|  | ≥14 g/day | 16.8 |  | 25 | 5 |  | 0.26 (0.08-0.81) |  |
| HSE/SHeSs § | Never drinker | 0.0 |  | 263 | 42 | HR | 1.00 (reference) | Age, sex, smoking, socioeconomic position, history of DM, hypertension, BMI, cholesterol-lowering medications, antihypertensive medications, antiplatelet agents, digoxin |
|  | Low-level drinker | 4.0 |  | 1630 | 307 |  | 0.81 (0.58–1.14) |  |
|  | Medium-level drinker | 28.0 |  | 458 | 73 |  | 0.76 (0.50–1.14) |  |
|  | High-level drinker | 85.1 |  | 68 | 11 |  | 0.85 (0.43–1.70) |  |
|  |  |  |  |  |  |  |  | ***(continued)*** |
| **First author, year** | **Alcohol consumption** | |  | **Risk of cardiovascular mortality** | | | | |
|  | **Reported exposure categories** | **Estimated g/day *†** |  | **Total (N)** | **Cases (n)** | **Measure of association** | **Effect estimates** | **Confounder adjustment** |
| UK Biobank § | Never drinker | 0.0 |  | 1076 | 54 | HR | 1.00 (reference) | Age, sex, smoking, education, history of DM, hypertension, BMI, cholesterol-lowering medications, antihypertensive medications, antiplatelet agents, digoxin, warfarin |
|  | Low-level drinker | 7.9 |  | 5988 | 219 |  | 0.65 (0.48–0.88) |  |
|  | Medium-level drinker | 30.6 |  | 5218 | 221 |  | 0.63 (0.46–0.86) |  |
|  | High-level drinker | 76.8 |  | 891 | 46 |  | 0.79 (0.53–1.19) |  |
| * The upper limit of the highest exposure category defined as the lower bound multiplied by 1.2, unless explicitly defined within each publication  † Average intake in each consumption category. Where unreported, the median of the upper and lower bounds was used  ‡ Effect estimates re-calculated according to a reference group other than that originally reported. This was undertaken using the Hamling method, as described in text  § Measures of usual weekly consumption are presented in line with the current UK guidelines, categorized as never drinker, low-level drinker (≤ 14 units/week), medium-level drinker ( >14 to ≤50 units/week for men, >14 to ≤35 units/week for women), or high-level drinker (>50 units/week for men, >35 units/week for women) | | | | | | | | |

# Table S5. Alcohol consumption, effect estimates, and confounder adjustment reported by studies on cardiovascular events

| **First author, year** |  | **Alcohol consumption** | |  | **Risk of cardiovascular events** | | | | |
| --- | --- | --- | --- | --- | --- | --- | --- | --- | --- |
|  |  | **Reported exposure categories** | **Estimated g/day *†** |  | **Total (N)** | **Cases (n)** | **Measure of association** | **Effect estimates** | **Confounder adjustment** |
| Levantesi, 2013 |  | Never/almost never | 0.0 |  | 4108 | 458 | HR | 1.00 (reference) | Age, gender, BMI, smoking, prior MI, history of hypertension, DM, peripheral vascular disease, electrical instability, exercise, LVEF, NYHA class, revascularization procedures, intakes of cooked vegetables, raw vegetables, fruit, fish, olive oil, other oil, butter, cheese, and coffee, use of n−3 PUFA, vitamin-E, antiplatelet agents, angiotensin-converting-enzyme inhibitor, lipid-lowering medication , beta-blockers |
|  |  | ≤0.5 L/day | 20.4 |  | 5446 | 551 |  | 0.87 (0.76–0.99) |  |
|  |  | >0.5 L/day | 49.0 |  | 1694 | 159 |  | 0.90 (0.74–1.09) |  |
| Masunaga, 2006 | Age < 65 years | Abstainers | 0.0 |  | 1385 | 54 | HR | 1.00 (reference) | CABG, atrial fibrillation, PCI, cholesterol-lowering agents, obesity, antiplatelet agents, β-blockers, warfarin, Forrester class, nitrates, coronary thrombolysis, calcium antagonists, DM, smoking, PVC, Gout, Killip class, ACE inhibitors, vasospastic angina, hyperlipidemia, multi-vessel disease, hypertension, positive exercise ECG, antiarrhythmic agents, angina pectoris |
|  |  | <30 ml/day | 11.9 |  | 1053 | 20 |  | 0.56 (0.32–0.97) |  |
|  |  | ≥30 ml/day | 28.4 |  | 563 | 18 |  | 0.92 (0.51–1.66) |  |
|  | Age ≥65 years | Abstainers | 0.0 |  | 533 | 24 | HR | 1.00 (reference) | Same as above |
|  |  | <30 ml/day | 11.9 |  | 250 | 14 |  | 1.02 (0.44–2.35) |  |
|  |  | ≥30 ml/day | 28.4 |  | 61 | 12 |  | 5.75 (2.21–14.90) |  |
| de Lorgeril, 2002 |  | Non-drinkers | 0.0 |  | 96 | 36 | RR | 1.00 (reference) | Diet group, age, current smoking, serum total cholesterol, and systolic blood pressure |
|  |  | <5.41% of total energy intake/day | 8.9 |  | 83 | 34 |  | 0.74 (0.40–1.38) |  |
|  |  | >5.41 but <9.84% | 24.1 |  | 89 | 18 |  | 0.41 (0.20–0.83) |  |
|  |  | >9.84% | 51.8 |  | 85 | 16 |  | 0.48 (0.24–0.96) |  |
| UK Biobank ‡ |  | Never drinker | 0.0 |  | 1076 | 258 | HR | 1.00 (reference) | Age, sex, smoking, education, history of DM, hypertension, BMI, cholesterol-lowering medications, antihypertensive medications, antiplatelet agents, digoxin, warfarin |
|  |  | Low-level drinker | 7.9 |  | 5989 | 1155 |  | 0.74 (0.64–0.85) |  |
|  |  | Medium-level drinker | 30.6 |  | 5218 | 1050 |  | 0.69 (0.60–0.80) |  |
|  |  | High-level drinker | 76.7 |  | 892 | 183 |  | 0.71 (0.58–0.86) |  |
| * The upper limit of the highest exposure category defined as the lower bound multiplied by 1.2, unless explicitly defined within each publication  † Average intake in each consumption category. Where unreported, the median of the upper and lower bounds was used  ‡ Measures of usual weekly consumption are presented in line with the current UK guidelines, categorized as never drinker, low-level drinker (≤ 14 units/week), medium-level drinker ( >14 to ≤50 units/week for men, >14 to ≤35 units/week for women), or high-level drinker (>50 units/week for men, >35 units/week for women) | | | | | | | | | |

# Figure S7. Dose-response relationship between alcohol consumption and risk of all-cause mortality, cardiovascular mortality, and cardiovascular events, stratified by sex

Best-fitting second-degree fractional polynomial models (with 95% CIs) are shown in solid curves with each data point overlaid as circles. Circle size indicates the weighting of each data point and is inversely proportional to the variance of the log-transformed relative risk.

a. All-cause mortality


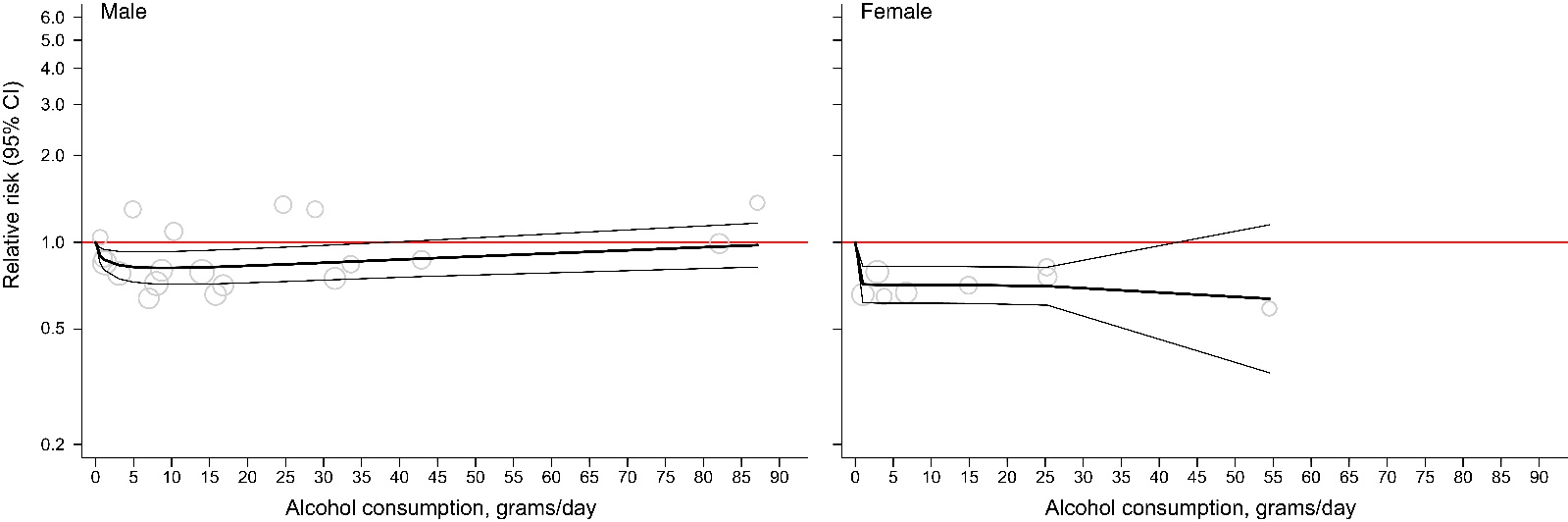


b. Cardiovascular mortality


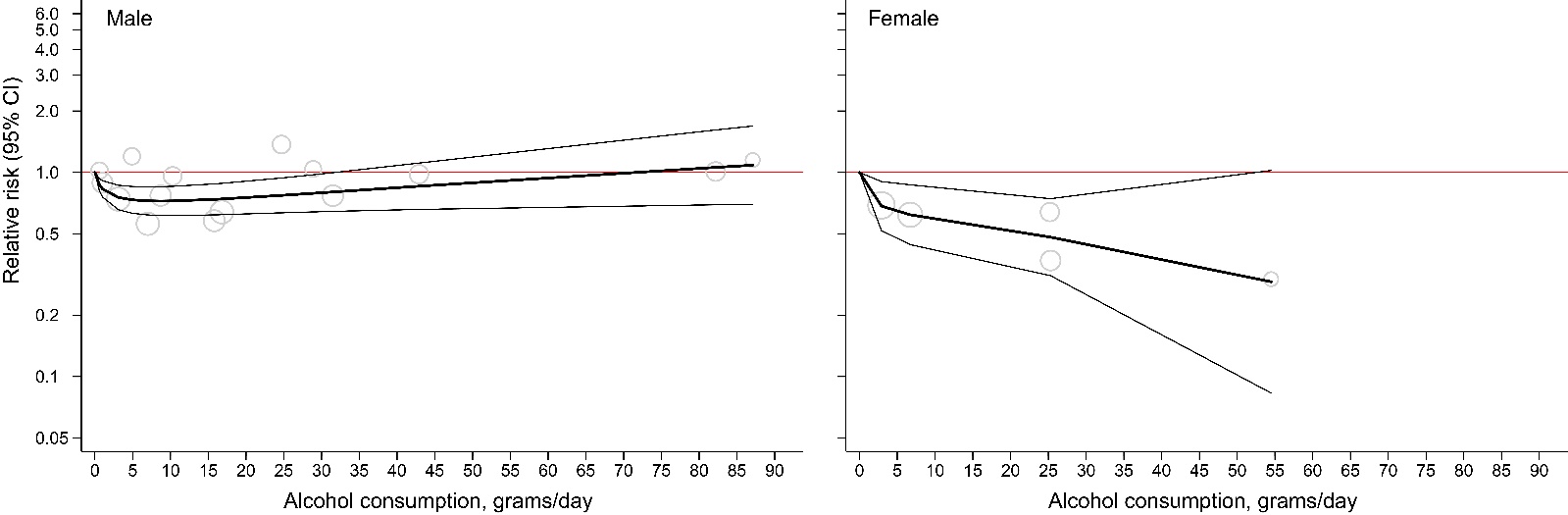


c. Cardiovascular events


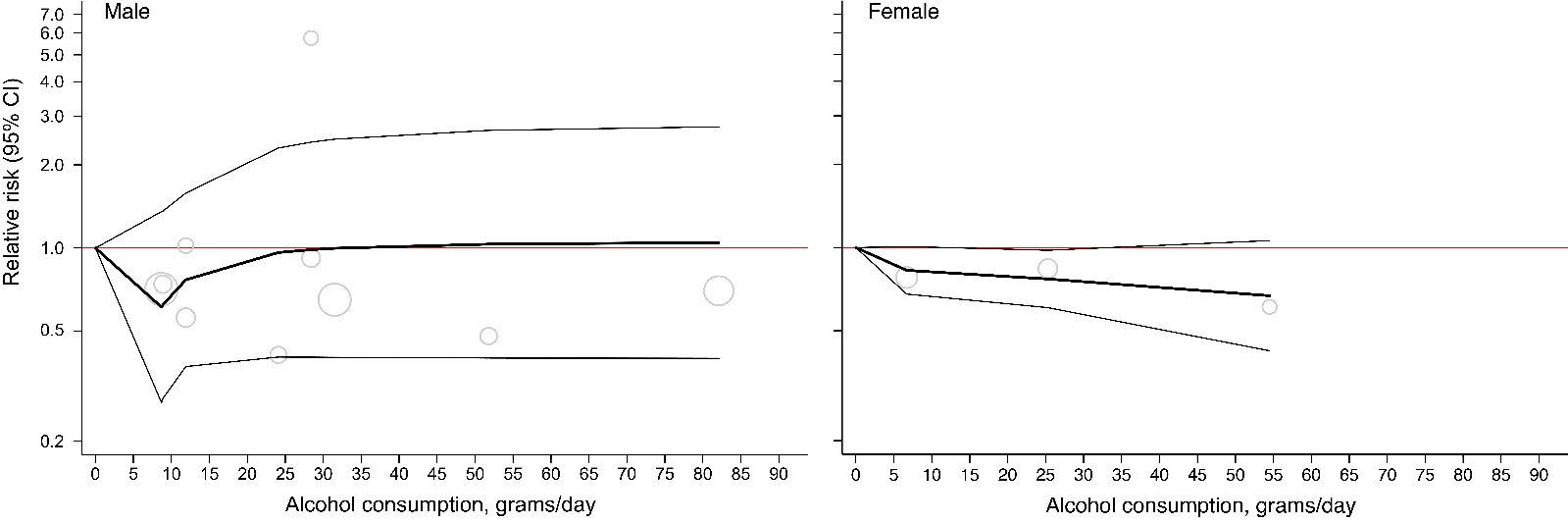


# Figure S8. Dose-response relationship between alcohol consumption and risk of all-cause mortality, cardiovascular mortality, and cardiovascular events, stratified by primary cardiovascular event

Best-fitting second-degree fractional polynomial models (with 95% CIs) are shown in solid curves with each data point overlaid as circles. Circle size indicates the weighting of each data point and is inversely proportional to the variance of the log-transformed relative risk.

a. All-cause mortality


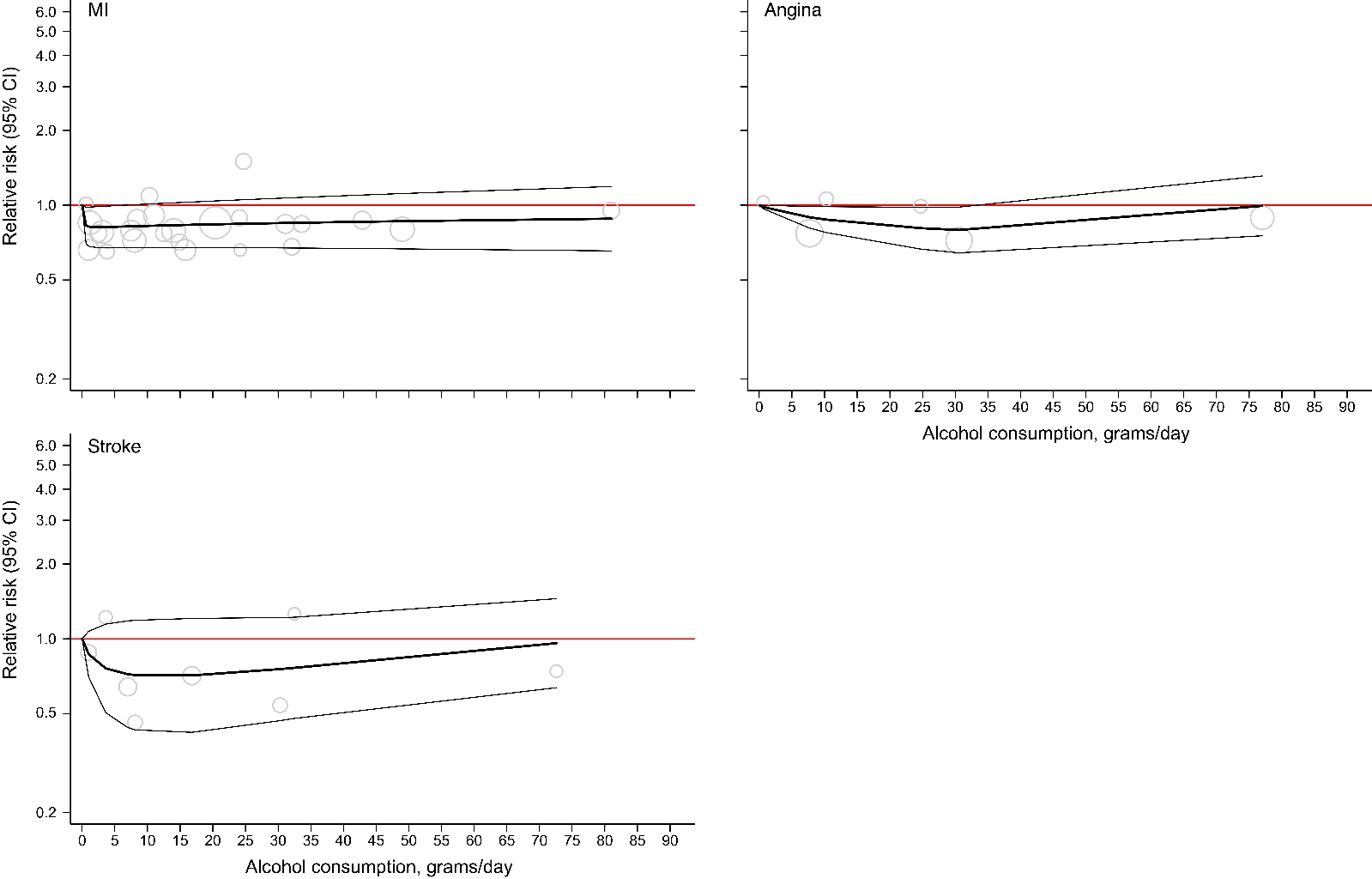


b. Cardiovascular mortality


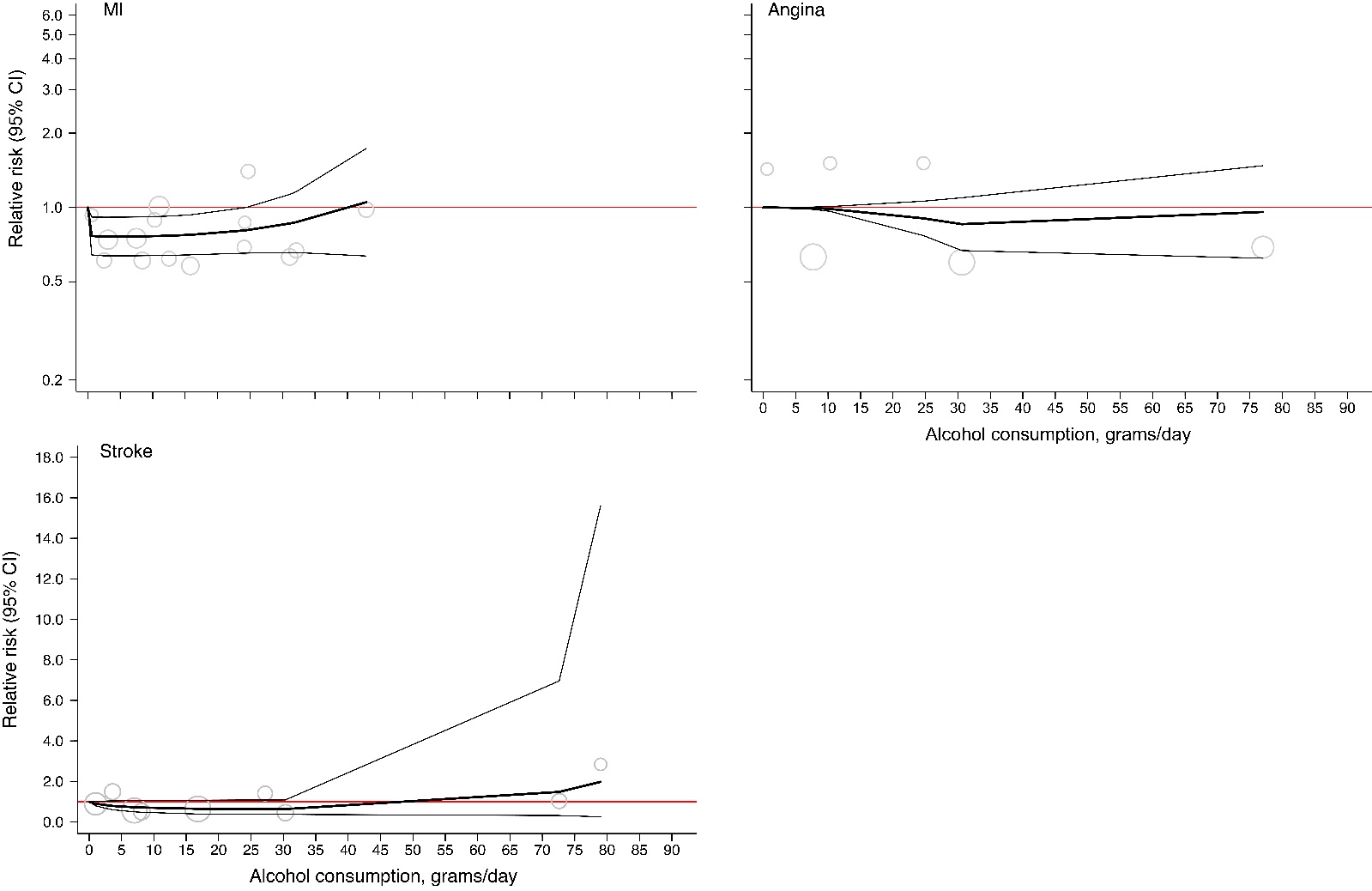


c. Cardiovascular events


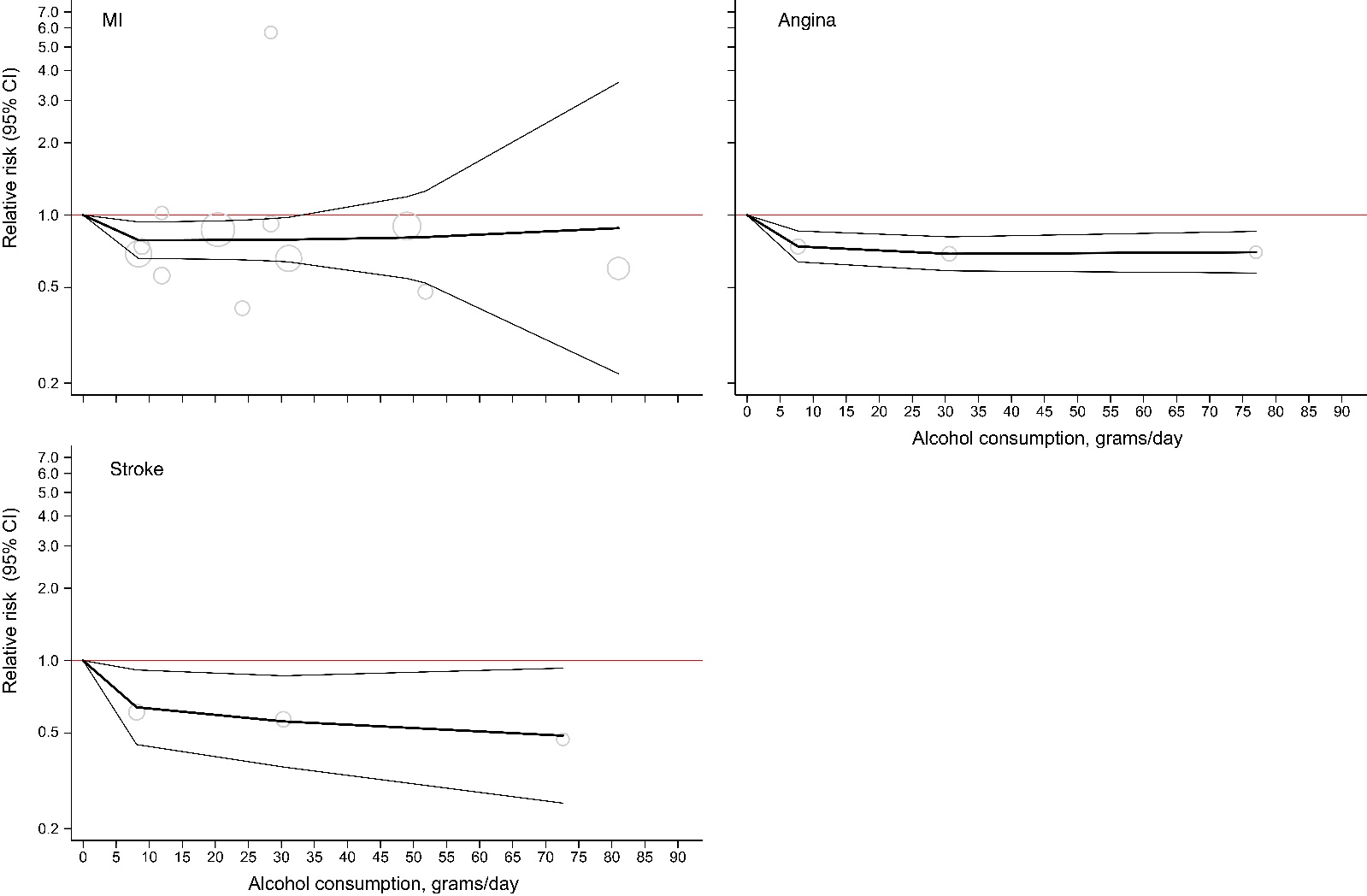


# Figure S9. Dose-response relationship between alcohol consumption and risk of all-cause mortality, cardiovascular mortality, and cardiovascular events, relative to different non-drinking reference group

Best-fitting second-degree fractional polynomial models (with 95% CIs) are shown in solid curves with each data point overlaid as circles. Circle size indicates the weighting of each data point and is inversely proportional to the variance of the log-transformed relative risk.

a. All-cause mortality


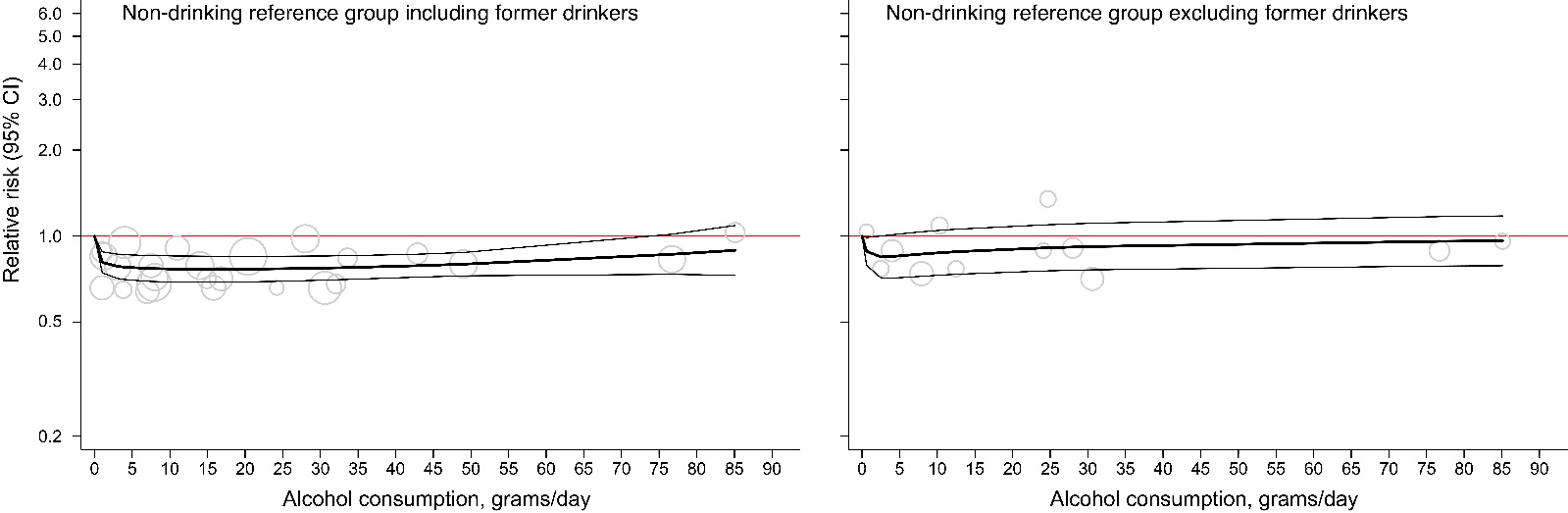


b. Cardiovascular mortality


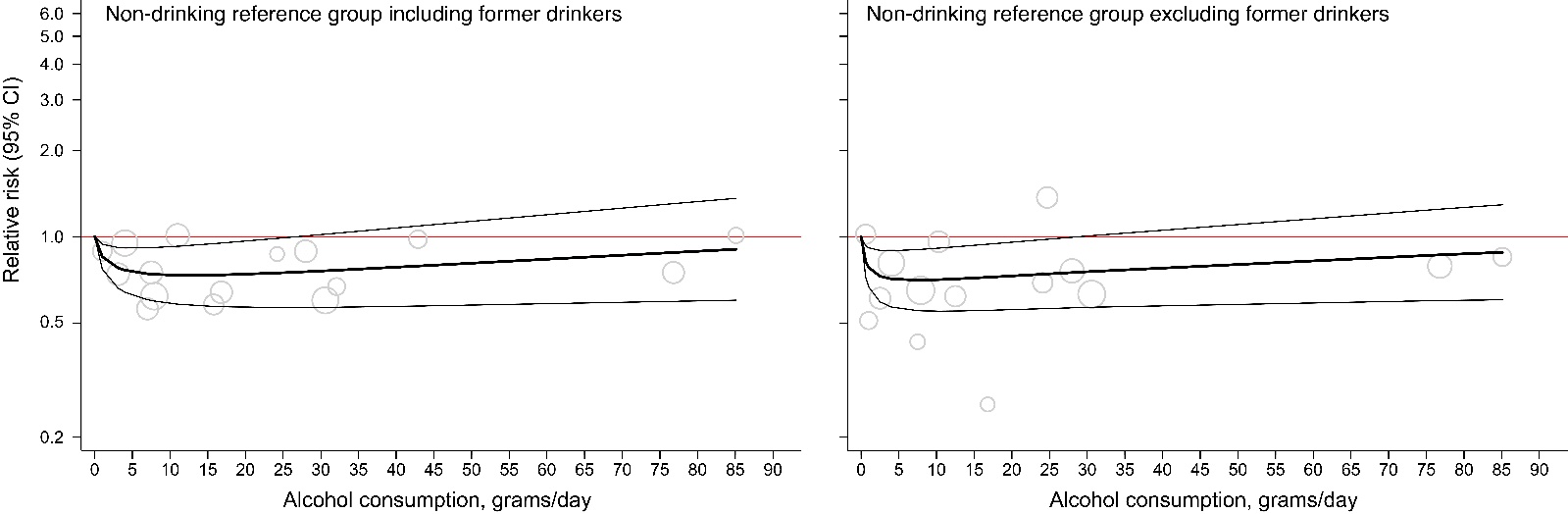


c. Cardiovascular events


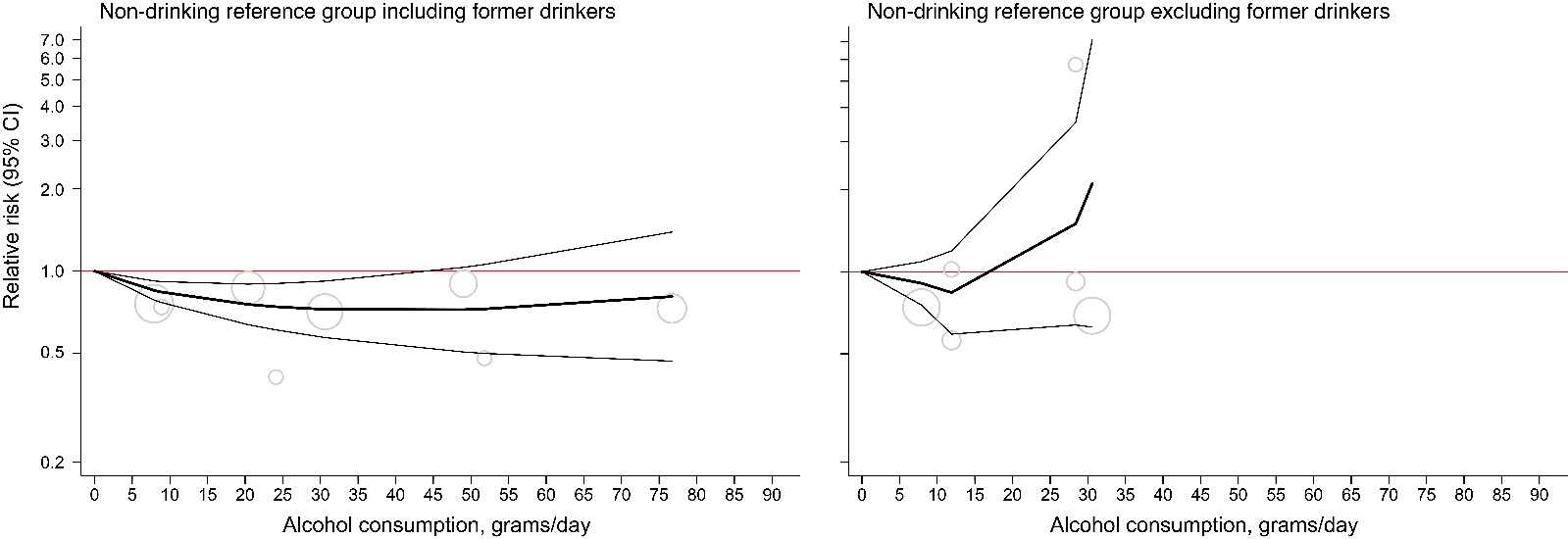


# Figure S10. Dose-response relationship between alcohol consumption and risk of all-cause mortality, cardiovascular mortality and cardiovascular events, using different method of assessing alcohol consumption

Best-fitting second-degree fractional polynomial models (with 95% CIs) are shown in solid curves with each data point overlaid as circles. Circle size indicates the weighting of each data point and is inversely proportional to the variance of the log-transformed relative risk.

a. All-cause mortality


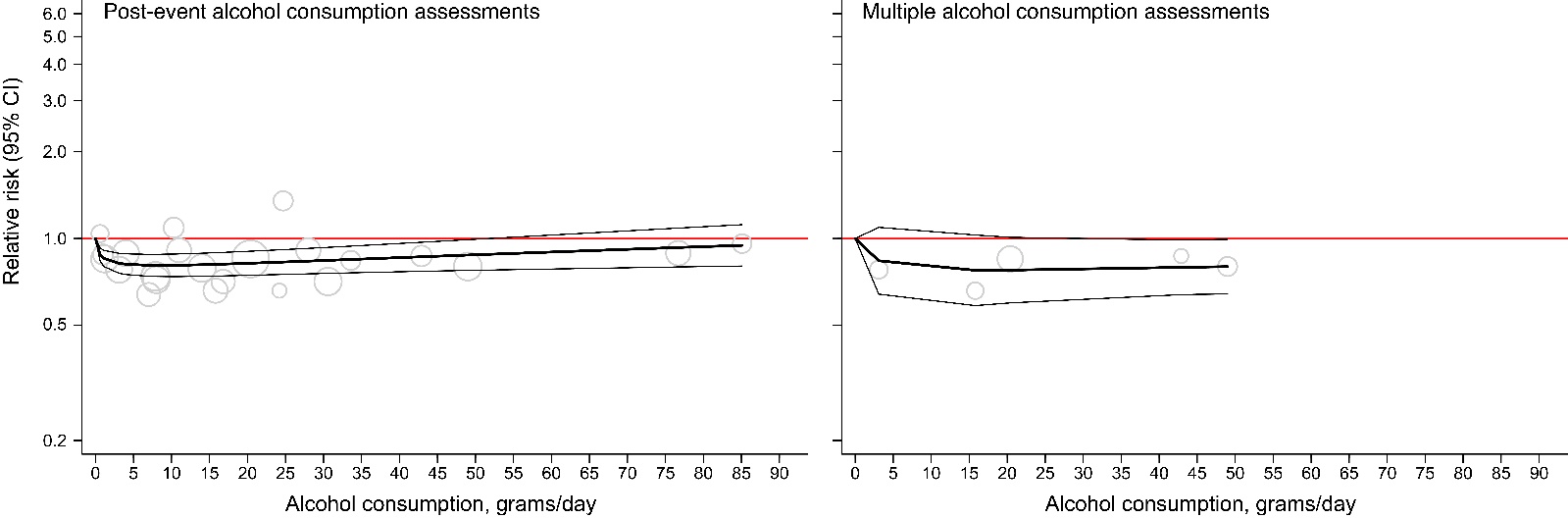


b. Cardiovascular mortality


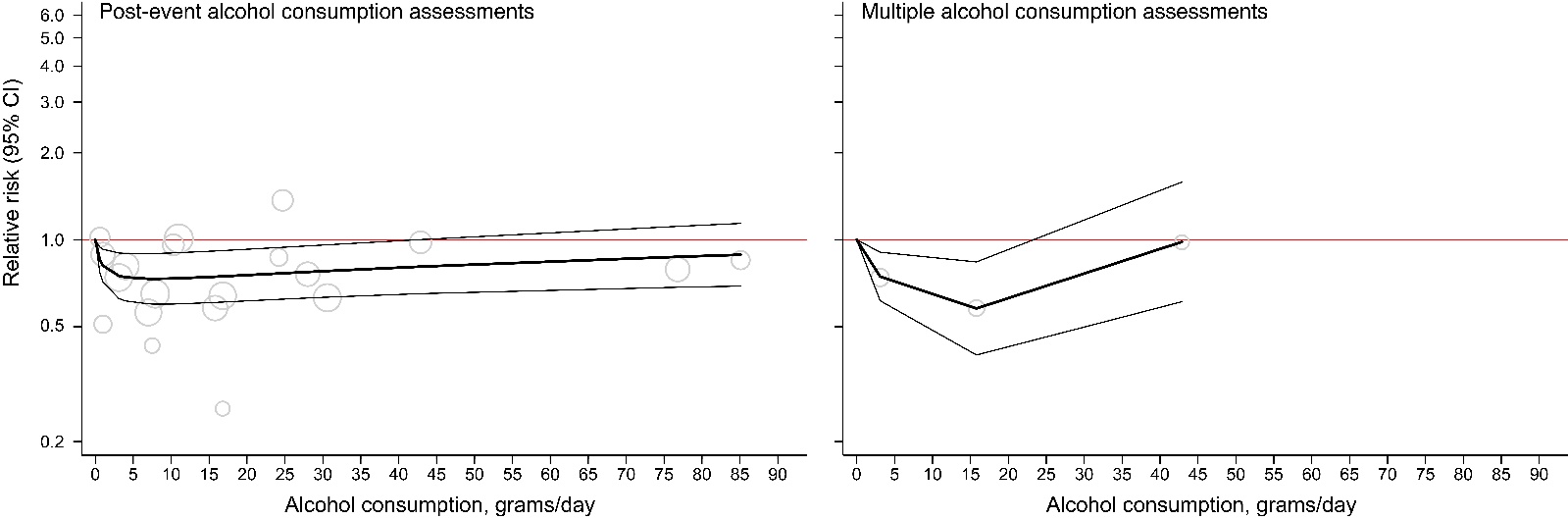


c. Cardiovascular events


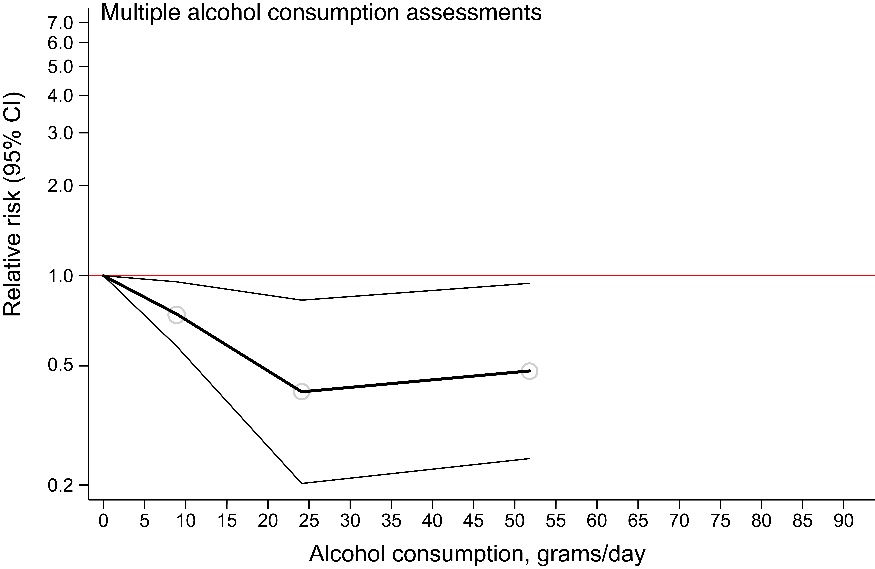


# Figure S11. Overall dose-response relationship between alcohol consumption and risk of all-cause and cardiovascular mortality after excluding studies with a quality assessment score <7

Best-fitting second-degree fractional polynomial models (with 95% CIs) are shown in solid curves with each data point overlaid as circles. Circle size indicates the weighting of each data point and is inversely proportional to the variance of the log-transformed relative risk.


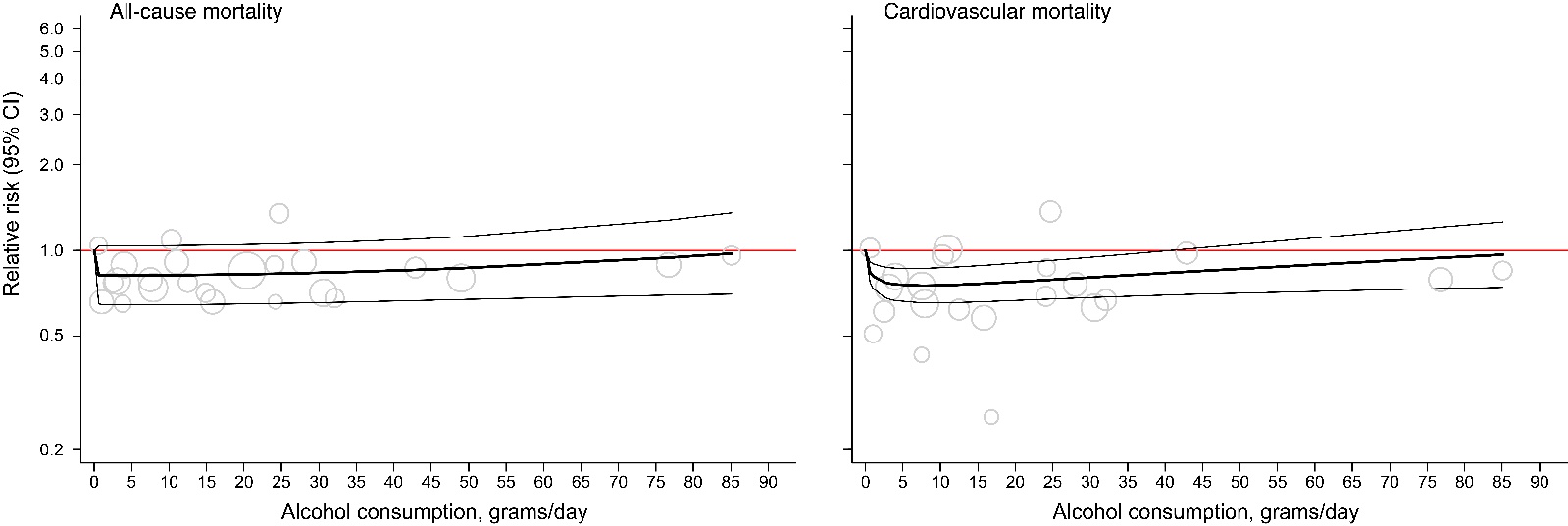


# Figure S12. Overall dose-response relationship between alcohol consumption and risk of all-cause mortality, cardiovascular mortality, and cardiovascular events, using least adjusted estimates (adjusted for age, sex, and smoking status only)

Best-fitting second-degree fractional polynomial models (with 95% CIs) are shown in solid curves with each data point overlaid as circles. Circle size indicates the weighting of each data point and is inversely proportional to the variance of the log-transformed relative risk.


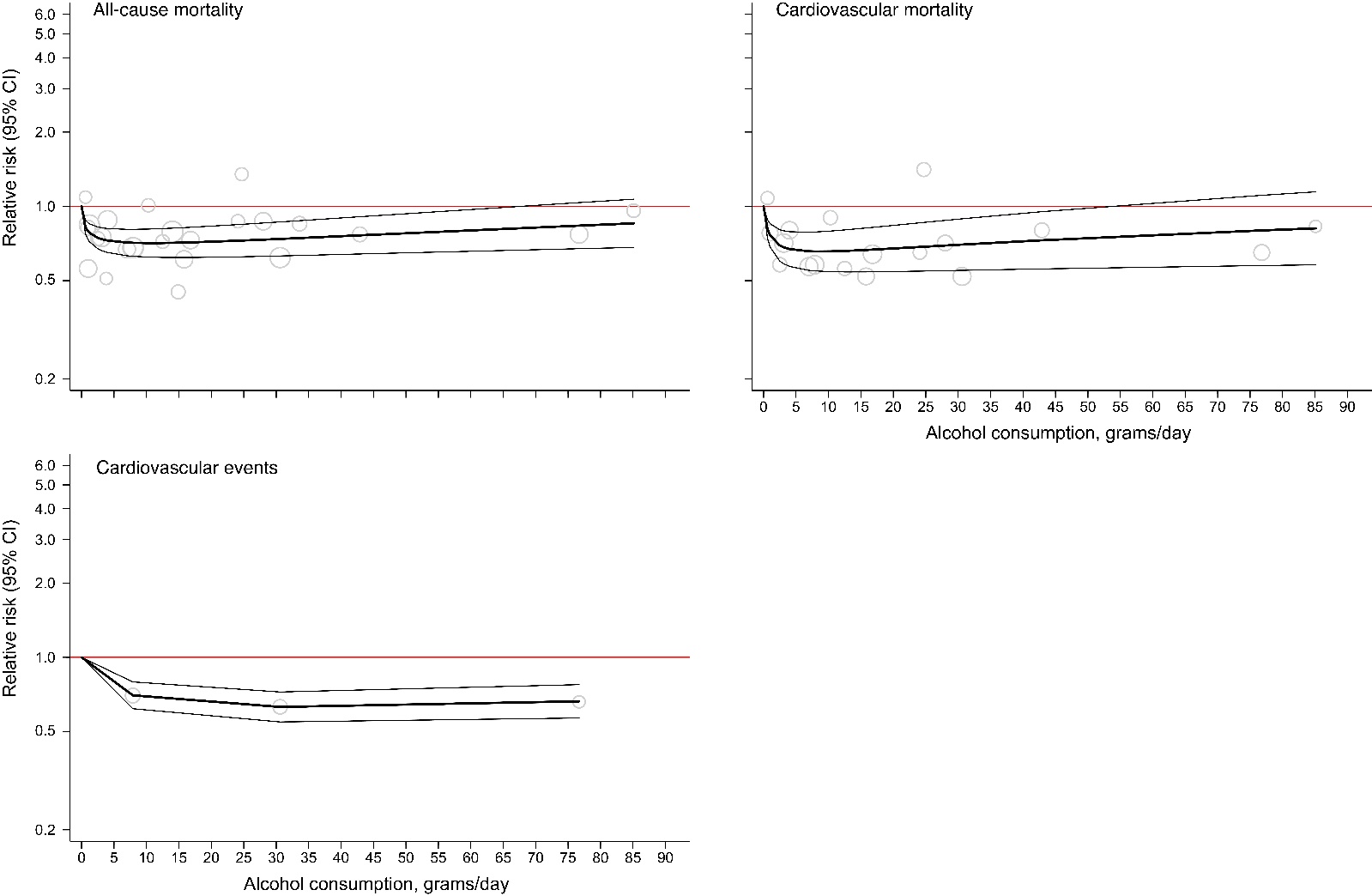


# Figure S13. Funnel plots

As asymmetry cannot be examined using continuous dose-response data, alcohol consumption in each study was reclassified into three groups (0-10 g/day, 10-20 g/day and >20 g/day) according to its averages of the reported categories. For each outcome, we then repeated our analysis for each drinking group.

a. All-cause mortality


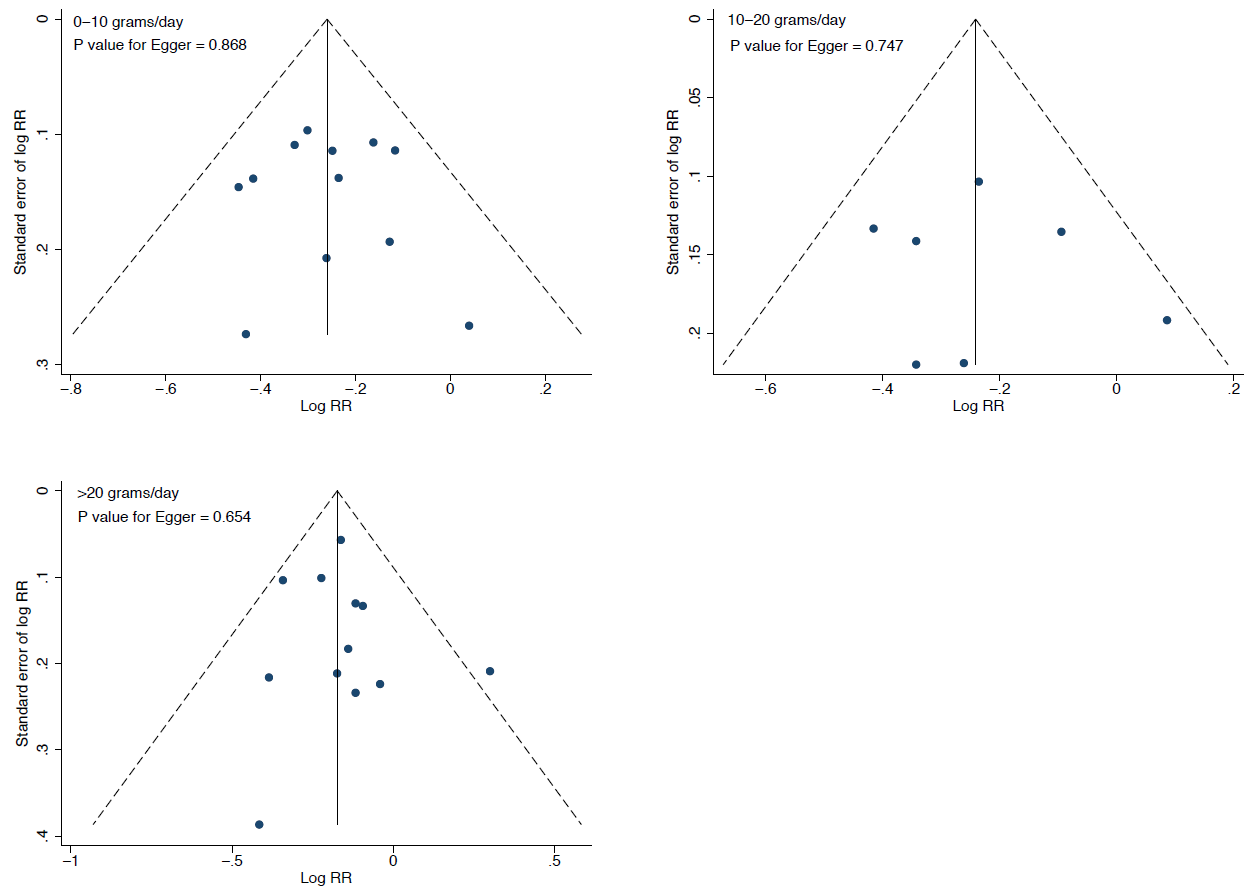


b. Cardiovascular mortality


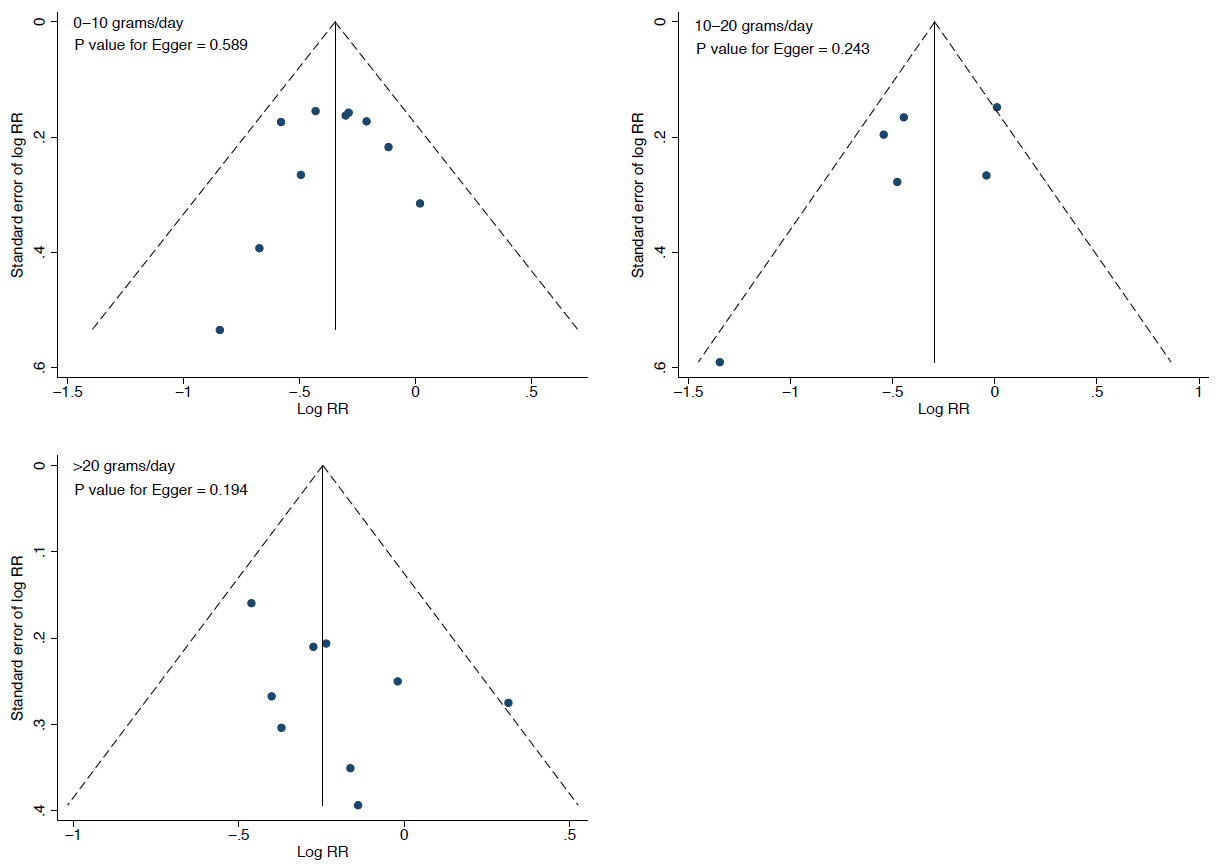


c. Cardiovascular events

***
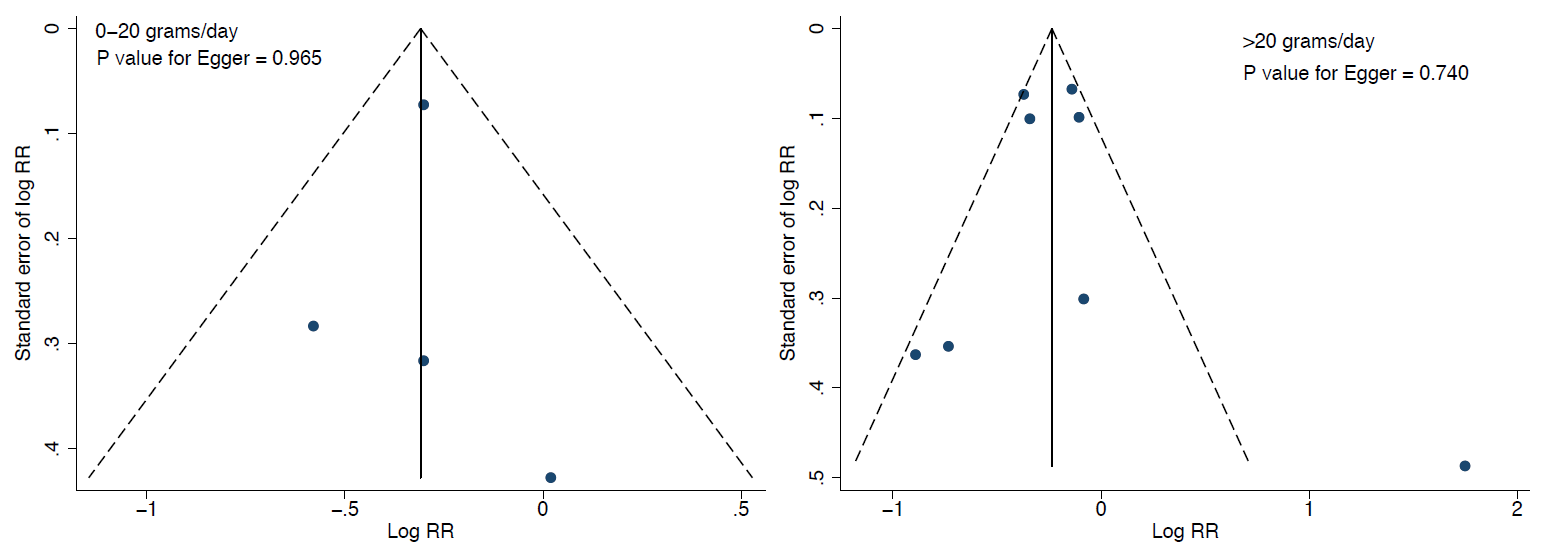
***

# Table S6. Associations of alcohol intake with HDL-cholesterol and gamma-glutamyl transferase in UK Biobank and HSE/SHeSs

| **Study cohort** | | **Alcohol consumption category** | | | | |  | **Alcohol intake (per 100 g/day) *** | |
| --- | --- | --- | --- | --- | --- | --- | --- | --- | --- |
|  |  | **Never drinker** | **Low-level drinker** | **Medium-level drinker** | **High-level drinker** | **Former drinker** |  | **β (95% CI)** | ***P*-value** |
| *UK Biobank* | |  |  |  |  |  |  |  |  |
| Gamma-glutamyl transferase (U/L) (N=13477) | | | |  |  |  |  |  |  |
|  | n | 1000 | 5611 | 4908 | 836 | 1122 |  |  |  |
|  | Mean (95% CI) † | 40.09 (36.46–43.72) | 41.79 (40.10–43.49) | 53.08 (51.16–54.99) | 79.75 (75.83–83.66) | 38.87 (35.46–42.27) |  | 59.11 (54.40–63.82) | <0.001 |
| *UK Biobank* | |  |  |  |  |  |  |  |  |
| HDL-cholesterol (mmol/L) (N=12334) | | |  |  |  |  |  |  |  |
|  | n | 917 | 5123 | 4481 | 766 | 1047 |  |  |  |
|  | Mean (95% CI) † | 1.19 (1.17–1.21) | 1.26 (1.25–1.27) | 1.35 (1.34–1.36) | 1.49 (1.47–1.51) | 1.20 (1.18–1.22) |  | 0.39 (0.36–0.41) | <0.001 |
| *HSE/SHeSs* | |  |  |  |  |  |  |  |  |
| HDL-cholesterol (mmol/L) (N=385) | | |  |  |  |  |  |  |  |
|  | n | 60 | 196 | 55 | 6 | 68 |  |  |  |
|  | Mean (95% CI) † | 1.24 (1.15–1.34) | 1.31 (1.26–1.37) | 1.43 (1.33–1.53) | 1.54 (1.25–1.83) | 1.26 (1.17–1.34) |  | 0.40 (0.14–0.67) | 0.003 |
| * β (95% CI) and *P*-values were derived from multivariable linear regression models by treating alcohol intake as a continuous variable  † Means (95% CI) were derived from multivariable linear regression models by treating alcohol consumption as a categorical variable  All models were adjusted for age, sex, smoking status, diabetes, hypertension, socioeconomic position or education, body mass index, cholesterol-lowering medications, antihypertensive medications, antiplatelet agents, digoxin, and warfarin  CI=confidence interval, HDL=high-density lipoprotein, HSE=the Health Survey for England, SHeSs=the Scottish Health Survey | | | | | | | | | |
